# Dissociable behavioural signatures of co-existing impulsivity and apathy in decision-making

**DOI:** 10.1038/s41598-022-25882-z

**Published:** 2022-12-12

**Authors:** Pierre Petitet, Sijia Zhao, Daniel Drew, Sanjay G. Manohar, Masud Husain

**Affiliations:** 1 Department of Experimental Psychology, University of Oxford, Oxford, OX1 3PH UK; 2 Centre de Recherche en Neurosciences de Lyon, Équipe Trajectoires, Inserm UMR-S 1028, CNRS UMR 5292, Université Lyon 1, Bron, France; 3 Nuffield Department of Clinical Neurosciences, University of Oxford, Oxford, OX3 9DU UK

**Keywords:** Motivation, Human behaviour, Motivation

## Abstract

Apathy and impulsivity are expressed in a wide range of neuropsychiatric disorders, and, to a less severe extent, in healthy people too. Although traditionally considered to be opposite extremes of a single motivational spectrum, recent epidemiological questionnaire-based data suggest that both traits can in fact co-exist within the same individual. Here, we sought to investigate the relationship between these constructs in healthy people within a controlled task environment that examines the ability to make a decision under temporal uncertainty and measures the vigour of the response. Sixty participants performed a new version of the Traffic Light Task and completed self-report questionnaire measures of apathy and impulsivity. The task required individuals to make rapid decision-making for time-sensitive reward by squeezing a hand-held dynamometer as quickly as possible after a predictable event occurred (a traffic light turning green). Although apathy and impulsivity were positively correlated in questionnaire assessments, the two traits were associated with distinct behavioural signatures on the task. Impulsivity was expressed as an inflexible tendency to generate rapid anticipatory responses, regardless of cost-benefit information. Apathy, on the other hand, was associated with a blunted effect of reward on response vigour. These findings reveal how apathy and impulsivity are related to distinct dimensions of goal-directed behaviour, explaining how these traits might co-exist in the same individuals.

## Introduction

A major goal of modern neuroscience is to understand the mechanisms underlying motivated behaviour, and their implementation in the brain. Disorders of motivation, such as apathy and impulsivity, offer a valuable opportunity to study such mechanisms when they become dysfunctional in clinical populations. The core defining features of these syndromes are the diminution of self-generated purposeful actions^1^ (apathy) and the tendency to decide/act quickly with little deliberation or forethought^2^ (impulsivity). These traits are observed across a wide range of neuropsychiatric disorders^3–5^ and, to a certain extent, in all individuals^6^. Although more precise definitions of these syndromes distinguish between various possible domains of expression^7–10^, it is broadly agreed that their genesis involves the disruption of key decision-making processes underlying motivated behaviour^11^. A perplexing finding across several recent reports is the positive correlation of apathy and impulsivity traits in both healthy individuals^6,12^ and patient^13–16^ groups. How can someone be both apathetic and impulsive?

Because they influence motivated behaviour in opposite directions (i.e., insufficient vs. excessive actions), apathy and impulsivity have often naturally been considered to be polar opposites. The best example of such framework is the *motivational spectrum hypothesis*, which posits that apathetic and impulsive behaviour may originate from opposite dysregulations of the same neural networks involved in motivated behaviour^11,17–21^. According to this view, motivated behaviour results from multiple cognitive processes (e.g., information seeking, option generation, option selection, cost-benefit decisions, action initiation, learning from outcome) that can each be directionally regulated to meet the specific constraints of the task environment. Ventral striatal dopamine is proposed to be the biological substrate that calibrates an individual’s behaviour along these multiple cognitive axes, with a net directional effect towards apathy or impulsivity depending on whether there is too little or too much dopamine^11^. Support for this model comes mostly from the literature on Parkinson’s disease, which contains descriptions of patients behaving apathetically or impulsively depending on whether they are in a hypo- or hyper- dopaminergic state, respectively^11,22–26^.

Implicit in the motivational spectrum hypothesis is the notion that, at any given time, an individual may behave either apathetically or impulsively, but crucially not both since these two states exist at opposite extremes of the same spectrum. A growing set of observations, however, appears to contradict this. Instances of co-occurrence of apathy and impulsivity have indeed been reported in patients suffering from Parkinson’s disease^14,16,25,27–29^, but also in frontotemporal lobar degeneration syndromes^13,15,30^, Alzheimer’s disease^31^, attention deficit hyperactivity disorders^32^, and schizophrenia^33^. In fact, even in individuals free from any neuropsychiatric diagnosis, the pattern of responses on self-report questionnaires reveals a certain degree of co-variation between the severity of apathetic and impulsive traits, especially within the goal-directed behavioural dimension^6,12^. Thus, apathy and impulsivity may derive not from opposite dysregulations of the same neural network, but instead from the disruption of distinct neural circuits that at least in part overlap^6,13^.

By challenging the motivational spectrum hypothesis, these observations open a range of new questions regarding the inter-relationship of apathy and impulsivity, the extent to which their respective mechanisms may overlap, and the ways in which they could be co-expressed. Can individuals act both impulsively and apathetically simultaneously, or do they instead fluctuate between these two behavioural states in response to environmental triggers (or lack thereof)? To our knowledge, the relationship between apathy and impulsivity has solely been explored in subjective self-report or clinician assessments (e.g., questionnaires or diagnostic criteria), and not in behavioural tasks. Even though both traits can be observed in isolation in dedicated tasks (e.g., apathy: Effort-Based Decision-Making task^34^, Option Generation task^35^; impulsivity: Balloon Analogue Risk-Taking task^36^, Cued Go No-Go task^37^), there is, to our knowledge, no experimental paradigm allowing for their potential co-expression.

In the present work, we aimed to investigate the mechanisms underlying the co-expression of apathy and impulsivity in healthy individuals within a single behavioural paradigm. To do so, we used a new version of the Traffic Light Task^38–40^ (TLT)—a task known to encourage impulsive behaviour—in order to incorporate potential opportunities for a co-expression with apathy. In the TLT, it is financially advantageous to take risks and behave in an anticipatory manner^38^. On each trial, a light successively turns red, amber then green, and participants are instructed to make a response as quickly as possible after the onset of the green “go” signal. Responses recorded prior to green onset incur a small monetary penalty, while those recorded after green onset are remunerated according to a reward function that declines at a rapid rate, so that within a few tens of milliseconds the reward that can be obtained is very low. Due to sensorimotor delays, standard human reaction times are typically much slower (few hundreds of milliseconds^41^) so it is impossible to collect the highest reward by merely reacting to the “go” signal. Instead, and because the temporal sequence of lights on TLT trials is sufficiently predictable though not fixed, participants can decide to initiate their response during the amber foreperiod in order to generate a response closer to the exact green onset. But in so doing they take a risk that their movement will actually be initiated before the light turns green, and thereby incur a small penalty. On the TLT, such anticipatory responses are still nevertheless advantageous in the long run because small penalties are worth incurring if many of the responses actually fall shortly after green onset, when the reward to be obtained is large.

To meet our study goals, the TLT was modified in three ways. First, the magnitude and decay rate of the reward function were manipulated to create different task environments in which it was more or less advantageous to anticipate. This allowed us to measure participants’ responsiveness to changes in the cost-benefit structure of the task environment, and determine whether such contextual adjustments could account for the apparent co-expression of apathy and impulsivity in self-report questionnaire data. For example, individuals who score high on both traits with standard assessments might in fact be particularly responsive to the environmental context, and thereby *fluctuate* to a greater extent between an apathetic and impulsive behavioural state in response to environmental triggers. Second, instead of an eye movement^39,40^ or a button press^38^, a hand-held dynamometer was used to record responses, such that they became physically effortful. This introduced an effort-based decision-making dimension to the task, a process known to be implicated robustly in apathy^34,42–45^. Lastly, the subjective perception of fatigue, which is also known to be associated with apathy^10,46–49^, offers an alternative route to explain an apparent co-expression between apathy and impulsivity. It might indeed be the case that impulsive individuals tend to build up more fatigue, which may secondarily promote apathy. To measure how fatigue developed in the TLT, subjective ratings of fatigue were collected at various times throughout the experiment.

Consistent with previous findings^6^, total apathy and impulsivity questionnaire scores were positively correlated in the healthy participants tested here ($n=60$). Nevertheless, when partialed out, the two constructs showed unique associations with distinct features of the modified TLT. Impulsivity was associated with a rigid urge to respond early, which was insensitive to changes in specific task constraints. Apathy, on the other hand, was associated with a blunted effect of reward on the energization of response vigour and on the prevention of fatigue. Finally, we found no evidence of an apathy-impulsivity axis on the TLT since the two traits appear to operate along distinct decision process components. Taken together, our results explain the possible co-expression of apathy and impulsivity in self-report questionnaire data: the two traits are in fact related to distinct dimensions of decision-making, which accounts for why they can be expressed in parallel in the same individual.

**Figure 1:**
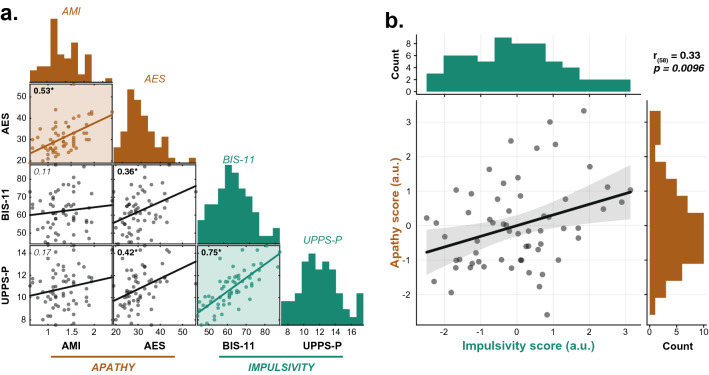
Self-report measures of apathy and impulsivity are positively correlated. (**a**) Pairwise correlation plot of the total scores of the four questionnaires. The strongest positive associations were observed between the two questionnaires assessing the same construct (Apathy: AES vs. AMI; Impulsivity: BIS-11 vs. UPPS-P; both $p<0.0001$). In addition, significant cross-construct correlations were observed between the AES and impulsivity measures (BIS-11 & UPPS-P; both $p<0.004$). Pearson correlation coefficients are shown in the top left corner of each scatter plot (asterisks indicate significant relationships, $p<0.05$). The sample distribution of each questionnaire total score is shown on top. (**b.**) PCAs were performed on apathy (AES & AMI total score) and impulsivity (BIS-11 & UPPS-P total scores) measures in order to capture, in a single score, most of the variance of the construct’s questionnaires. These two scores were positively correlated ($r_{(58)}=0.33$, $p=0.0096$).

## Results

### Self-report measures of apathy and impulsivity are positively correlated

On the day of the experimental session, participants ($n=60$; 30 females; mean age: 24.97 years, *s.d.*: 4.85) filled out two validated self-report questionnaire measures of impulsivity (Barratt Impulsiveness Scale, BIS-11^8^ Urgency, Premeditation (lack of), Perseverance (lack of), Sensation Seeking, Positive Urgency, Impulsive Behavior Scale, UPPS-P^9^) and two measures of apathy (Apathy Evaluation Scale, AES^7^; Apathy Motivation Index^10^). Consistent with previous observations in healthy individuals using these same measures^6^, positive correlations were observed within and across the two constructs assessed by these questionnaires (Fig. 1a). For each construct, a Principal Component Analysis (PCA) was performed to derive a global index (PCA score) quantifying its severity across the two self-report questionnaires used to assess it (Apathy score: PCA of AES & AMI total scores; Impulsivity score: PCA of BIS-11 & UPPS-P total scores; see *Methods*). As expected, these two scores positively correlated with one another ($r_{(58)}=0.33$, $p=0.0096$; Fig. 1b) such that individuals who reported being overall more impulsive also reported being more apathetic. In order to identify behavioural markers uniquely associated with apathy and/or impulsivity, all subsequent questionnaire-based analyses systematically included both these PCA scores within the same regression model (partial regressions).

### Experimental paradigm

**Figure 2:**
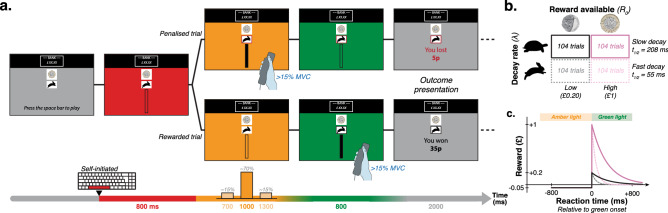
Overview of the Traffic Light Task. (**a**) A picture of a coin ($\pounds$1, $\pounds$0.20) and an animal (turtle, hare) indicated the reward magnitude and decay rate for the upcoming trial. Pressing the space bar initiated the sequence of red, amber, green background colours. The duration of the amber light was either 700 ms (15.38% of trials), 1000 ms (69.23% of trials) or 1300 ms (15.38% of trials). Participants won monetary reward by squeezing a hand-held dynamometer above 15% of the Maximum Voluntary Contraction (MVC) as quickly as they could after the background turned green. (**b**) Both the intercept ($R_0$) and decay rate ($\lambda$) of the exponential reward function were experimentally manipulated in a two-by-two design (104 trials per condition in total, uniformly spread across eight experimental blocks). (**c**) Visual representation of the reward function of the four experimental conditions.

The present study used a new version of an established measure of rapid decision-making for reward, the Traffic Light Task^38–40^ (TLT). In our paradigm, a visual display successively turned red (800 ms), amber (700 ms on $15\%$ of trials, 1000 ms on $70\%$ of trials, 1300 ms on $15\%$ of trials) then green (800 ms), and participants had to gently squeeze a hand-held dynamometer (above 15% of their Maximum Voluntary Contraction, MVC) after the green onset in order to receive time-sensitive monetary reward (Fig. 2a). Anticipatory responses (i.e., supra-threshold squeezes recorded before the green onset) led to a small penalty ($\pounds$0.05). Conversely, responses recorded after the green onset were rewarded according to the following rapid exponential discounting function (Fig. 2c):

1 $$\begin{aligned} {\left\{ \begin{array}{ll} &{}R(RT)\ =\ R_0 \times e^{-\lambda \times RT} \ \ \ \text {if} \ RT\ge 0\\ &{}R(RT)\ = -0.05 \ \ \ \text {if} \ RT<0 \end{array}\right. } \end{aligned}$$

where R is the reward obtained as a function of the reaction time relative to the green onset, RT. Both the reward available ($R_0$) and decay rate after green onset ($\lambda$) were manipulated in a two-by-two design (Fig. 2b).

### Anticipation but not vigour leads to larger earning

Similar to previous versions of the task, it was functionally advantageous to respond early, as participants who made more penalised anticipatory responses nevertheless accumulated more reward overall ($\beta = 0.73$, $95\%$ CI = [0.57, 0.90], $t_{(57)}=8.70$, $p<0.001$, controlling for mean vigour; LMM S1; Table S1—Model 1; Fig. S1). By contrast, and by design, the vigour of the response (i.e., maximum contraction generated during the response, expressed as a percentage of the MVC) showed no instrumental relationship to earnings ($\beta = 0.13$, $95\%$ CI = $[-0.34, 0.30]$, $t_{(57)}=1.55$, $p=0.13$, controlling for percentage of anticipatory responses; contrast anticipatory responses vs. mean force: $F_{(1)}=22.90$, $p<0.001$). A within-participant trialwise analysis of the determinants of the reward obtained (LMM S2) also demonstrated that although trials in which individuals responded faster led to greater outcomes ($\beta = -0.08$, $95\%\,\hbox {CI} = [-0.11, -0.05]$, $t_{(24685)}=-5.52$, $p<0.001$), the amount of force exerted on the dynamometer was not associated with the reward obtained ($\beta = 0.01$, $95\%\,\hbox {CI} = [-0.01, 0.02]$, $t_{(24685)}=0.27$, $p=0.27$). This is consistent with the absence of significant correlation between response vigour and reaction time in the task (LMM S3 ; $\beta = 0.01$, $95\%\,\hbox {CI} = [-0.06, 0.08]$, $t_{(24689)}=0.34$, $p=0.73$). The use of a hand-held dynamometer therefore introduced a physical effort dimension to the task that was not directly rewarded because it did not straightforwardly translate to faster responses.

### Impulsivity but not apathy is advantageous on the TLT

Given that our version of the TLT provided a task environment in which it was financially advantageous to respond early (Fig. S1), we hypothesised that individuals who are naturally more impulsive may have a functional advantage. That is, the questionnaire-derived impulsivity score might positively correlate with total earnings. Similarly, if apathy operates along the same decision-making axis but has an opposite influence (motivational spectrum hypothesis), then it may be negatively associated with earnings (i.e., more apathetic people might win less reward overall).

To test these hypotheses, a single linear mixed-effect model (LMM) was built with both questionnaire-derived scores as predictors of total earning (LMM S4), controlling for age (fixed effect) and gender (random effect). The results showed that individuals with higher impulsivity scores indeed accumulated more reward overall on the task ($\beta = 0.29$, $95\%\,\hbox {CI} = [0.03, 0.54]$, $t_{(56)}=2.26$, $p=0.027$; Table S1—Model 2). The severity of apathy, by contrast, showed no significant relationship to earnings ($\beta = -0.09$, $95\%\,\hbox {CI} = [-0.45, 0.26]$, $t_{(56)}=-0.54$, $p=0.59$; Table S1—Model 2). In other words, although it was advantageous to be impulsive in our paradigm, it was neither advantageous nor disadvantageous to be apathetic. This is one line of evidence that apathy is not merely the negative counterpart of impulsivity, but instead might operate via distinct, dissociable, behavioural channels.

### Impulsivity is associated with a rigid all-encompassing urge to respond early

Next, we set out to characterise the specific means by which impulsivity conferred a functional advantage on the TLT. Were the most impulsive individuals merely faster to respond overall and/or did they strategically tailor their fast responses to the specific conditions that would yield highest reward? In order to answer this question, we first needed to establish which experimental factors influenced the speed of the response on our modified TLT. Five experimental factors were considered:

- The amber light duration of the *current* trial, $a_{n}$ (700, 1000 or 1300 ms);
- The amber light duration of the *previous* trial, $a_{n-1}$ (700, 1000 or 1300 ms);
- Task practice, captured as the block index (1, 2, 3, 4, 5, 6, 7, or 8);
- The reward available, $R_0$ (low: $\pounds$0.20; high: $\pounds$1);
- The exponential decay rate of reward, $\lambda$ (slow: 0.0033; fast: 0.0125).

**Figure 3:**
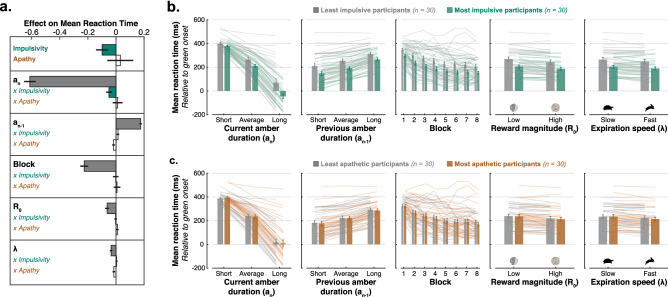
Impulsivity is associated with an all-encompassing pre-emptiveness to respond. (**a**) Parameter estimates of the linear mixed-effect model of mean RT (model controlling for Age and Gender; see Table S2—Model 2). Coloured bars show significant effects ($p<0.05$) and white bars non significant effects with standard error. Five experimental factors influenced the speed of the response: duration of the amber light of the current ($a_{n}$) and previous ($a_{n-1}$) trial, block index ($\log$-transformed), magnitude ($R_0$) and decay rate ($\lambda$) of the reward. Impulsivity had a significant negative main effect and interaction with $a_{n}$. This behavioural signature reflects a rigid tendency to respond early, regardless of the experimental condition. (**b–c.**) For visualisation purposes, the average reaction time (± *s.e.m.*) is plotted as a function of the five experimental factors of interest, with a group median split on the impulsivity score (**b**) or apathy score (**c**). Individual data are plotted as separate lines of the corresponding colour.

The main effect of these five factors was assessed using a single LMM with conditional average RT (relative to green onset) as the dependent variable and participant as a random effect (LMM S5; Fig. 3). Block index was $\log$-transformed as this proved to yield a better fit to the data ($\Delta BIC = -241.62$) and was consistent with an exponential learning curve, a common feature of human behaviour (e.g., reinforcement learning^50^, sensorimotor learning^51^). All explanatory variables were z-scored to obtain standardised parameter estimates.

The amber light duration of both the current and previous trials significantly influenced mean RT (Main effect of $a_n$ on mean RT: $\beta = -0.62$, $95\%\,\hbox {CI} = [-0.67, -0.57]$, $t_{(9294)}=-22.59$, $p<0.001$; Main effect of $a_{n-1}$ on mean RT: $\beta = 0.18$, $95\%\,\hbox {CI} = [0.16, 0.19]$, $t_{(9294)}=19.51$, $p<0.001$; Table S2—Model 1). Participants responded faster when the current amber light lasted longer than usual, and slower when it lasted shorter than usual (Fig. 3). By contrast, they responded faster when the previous amber light lasted shorter than usual, and slower when the previous amber light lasted shorter than usual (Fig. 3). These two effects illustrate two sides of the same process: the fact that participants built an internal estimate of the duration of the amber light, which they used to anticipate the green light onset (sensitivity to $a_n$), and updated on a trial-by-trial basis (sensitivity to $a_{n-1}$). These effects were also observed in previous versions of the task^38–40^, and are regarded as key features of the TLT.

Participants improved markedly (i.e., they learnt to respond earlier) with task practice. This was expressed as a negative effect of block index ($\log$-transformed) on mean RT ($\beta = -0.23$, $95\%$ CI = $[-0.27, -0.19]$, $t_{(9294)}=-12.06$, $p<0.001$; Table S2—Model 1; Fig. 3). Follow-up pairwise t-test comparisons of block averaged RTs showed that performance reached an asymptotic level in the fifth block as blocks 1 to 4 significantly differed from all others (all $p < 0.001$, Bonferroni-corrected) but it was no longer the case from the fifth block onwards. This learning effect appeared to result from a shift in strategy—from reaction to anticipation—rather than mere predictive learning based on the statistical properties of the task. The latter—assessed with a separate “Predictive” control task interleaved between TLT blocks (see *Methods*)—behaved very differently, plateauing much earlier during the experimental session (see *Supplementary Results*; Fig. S3).

Importantly, both the reward available ($R_0$) and the rate of its decay after green onset ($\lambda$) influenced the average RT. Participants responded faster on average when more reward was available (Main effect of $R_0$ on mean RT: $\beta =-0.06$, $95\%\,\hbox {CI} = [-0.08, -0.04]$, $t_{(9294)}=-6.12$, $p<0.001$; Table S2—Model 1; Fig. 3), and when reward decayed faster after the green onset (Main effect of $\lambda$ on mean RT: $\beta =-0.04$, $95\%\,\hbox {CI} = [-0.05, -0.02]$, $t_{(9294)}=-3.82$, $p<0.001$; Table S2—Model 1; Fig. 3). Thus, individuals were incentivised to respond earlier both to harvest larger amounts of reward, and to avoid losing too much of it. There was no significant interaction between the two factors ($\beta =-0.01$, $95\%\,\hbox {CI} = [-0.02, 0.00]$, $t_{(9294)}=-1.68$, $p=0.09$; Table S2—Model 1).

Having established that the average speed of the response on the TLT was influenced by task statistics ($a_n$, $a_{n-1}$), learning (block), and cost-benefit environment ($R_0$, $\lambda$), the key question was to determine whether the functional advantage enjoyed by impulsive individuals involved any of these factors. To answer this, we added the apathy and impulsivity scores and their interactions with experimental factors to the LMM reported above, while controlling for age (fixed effect) and gender (random effect; LMM S6). This revealed that more impulsive individuals responded generally earlier, regardless of the specific task constraints (Negative main effect of impulsivity score on mean RT: $\beta = -0.10$, $95\%\,\hbox {CI} = [-0.18, -0.01]$, $t_{(9282)}=-2.28$, $p=0.023$; Table S2—Model 2; Fig. 3a–b). In addition, their average RT was more influenced by the duration of the amber light of the current trial (Interaction Impulsivity score $\times$ $a_n$: $\beta = -0.05$, $95\%\,\hbox {CI} = [-0.09, -0.01]$, $t_{(9282)}=-2.46$, $p=0.014$; Table S2—Model 2; Fig. 3a–b). This suggests that more impulsive individuals were faster not because of shorter sensorimotor delays (see also *Supplementary Results* for an absence of relationship between impulsivity and simple reaction time) but instead because of a greater tendency to anticipate the green onset. None of the other interactions with experimental factors were significant for neither impulsivity nor apathy (Table S2—Model 2; Fig. 3a).

Taken together, these results are consistent with the functional advantage of impulsivity on the TLT deriving from an urge to respond earlier rather than any strategic adjustment to specific task constraints. Follow-up parcellation of this result into the individual sub-scales of the UPPS-P questionnaire revealed that it was driven significantly by positive urgency (Negative main effect of UPPS-P “Positive Urgency” on mean RT: $\beta = -0.13$, $95\%\,\hbox {CI} = [-0.25, -0.01]$, $t_{(9281)}=-2.06$, $p=0.039$; Interaction UPPS-P “Positive Urgency’ $\times$ $a_n$: $\beta = -0.06$, $95\%\,\hbox {CI} = [-0.12, -0.00]$, $t_{(9281)}=-2.05$, $p=0.041$; Table S3). Furthermore, *Supplementary Results* provide a computational parameterisation of this finding within a variant of a two-horse linear rise-to-threshold model^38–40^ able to reproduce the full RT distribution (Figs. S4-S8). The main conclusion of this alternative analysis is that more impulsive individuals showed a greater baseline probability to activate the anticipatory process in the task. This is consistent with the results of the simpler analysis reported in the main text.

### Apathetic individuals are less energized by reward

The use of a hand-held dynamometer in our modified TLT enabled us to measure not only the speed of the response but also its vigour (i.e., peak force exerted within each trial). This metric has been proposed to offer an indirect read-out of utility^52^, i.e., the subjective value individuals attribute to particular actions/stimuli. The force requirements to trigger a response were voluntarily chosen to be low (15% of the MVC) to avoid exhaustion, and leave room for potential overshoot. On average, participants overshot this threshold by $39.71\%$ (s.d.: $19.30\%$).

The same analysis strategy as the previous section was adopted, with the difference that the LMM additionally controlled for mean RT to orthogonalise vigour and RT (LMM S7). Before investigating interactions with apathy and impulsivity, we first established which of the five experimental factors ($a_n$, $a_{n-1}$, block index, $R_0$, $\lambda$) influenced the vigour of the response. The duration of the amber light of the current but not previous trial had an influence on the vigour of the response (Main effect of $a_n$ on mean vigour: $\beta = -0.10$, $95\%\,\hbox {CI} = [-0.12, -0.07]$, $t_{(9293)}=-7.48$, $p<0.001$; Main effect of $a_{n-1}$ on mean vigour: $\beta = 0.00$, $95\%\,\hbox {CI} = [-0.01, 0.01]$, $t_{(9293)}=-0.17$, $p=0.87$; Table S4—Model 1). Thus, the surprise induced by a exceptionally short amber light duration was reflected in a harder squeeze than usual, while the opposite was true for a longer amber foreperiod. This surprise effect, however, led to no force adjustment from one trial to the next (no significant effect of $a_{n-1}$). As the task progressed, the vigour of the response diminished (Main effect of $\log$-transformed block on mean vigour: $\beta = -0.19$, $95\%\,\hbox {CI} = [-0.24, -0.13]$, $t_{(9293)}=-6.24$, $p<0.001$; Table S4—Model 1), likely due to a fatigue effect (see *Supplementary Results* for an analysis of the relationship with fatigue ratings) and/or a better estimation of force threshold required to trigger a response.

**Figure 4:**
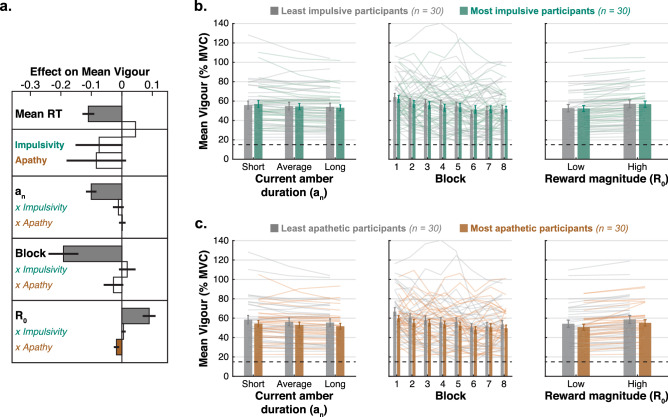
Apathy is associated with blunted valuation of reward. (**a**) Parameter estimates of the linear mixed-effect model of mean Vigour (model controlling for Age and Gender; see Table S4—Model 2). Coloured bars show significant effects ($p<0.05$) and white bars non significant effects with standard error. Three experimental factors influenced the speed of the response: duration of the amber light of the current trial ($a_{n}$), block index ($\log$-transformed), and magnitude of the reward available ($R_0$). Apathy negatively interacted with the latter effect. (**b–c**) For visualisation purposes, the average vigour (± *s.e.m.*) is plotted as a function of the three experimental factors of interest that significantly influenced it, with a group median split on the impulsivity score (**b**) or apathy score (**c**). Individual data are plotted as separate lines of the corresponding colour. The force requirement to trigger a response ($15\%$ MVC) is shown as a horizontal dotted black line.

Finally, and crucially, participants were energized to squeeze harder when more reward ($\pounds$1 vs. $\pounds$0.20) was available (Main effect of $R_0$ on mean vigour: $\beta = 0.09$, $95\%\,\hbox {CI} = [0.07, 0.11]$, $t_{(9293)}=8.68$, $p<0.001$; Table S4—Model 1), an effect we interpreted as a marker of reward sensitivity. By contrast, the decay rate of reward or the interaction between these two factors had no significant influence on the vigour of the response (Main effect of $\lambda$ on mean vigour: $\beta = -0.01$, $95\%\,\hbox {CI} = [-0.03, 0.01]$, $t_{(9293)}=-0.61$, $p=0.54$; Interaction $R_0 \times \lambda$: $\beta = 0.00$, $95\%\,\hbox {CI} = [-0.01, 0.01]$, $t_{(9293)}=0.20$, $p=0.84$; Table S4—Model 1). Thus, in summary, the vigour of the response was influenced by: (1) surprising amber foreperiod duration, (2) task-practice, and (3) the magnitude of reward available (Fig. 4). It can therefore be seen as a composite measure that partly captures surprise, sensorimotor learning and subjective utility.

Next, we asked whether apathy or impulsivity interacted with any of these three effects. Adding the questionnaire-derived scores to the LMM (LMM S8) revealed that the severity of apathy was associated with a weaker energization by reward (Interaction Apathy score $\times$ $R_0$: $\beta = -0.02$, $95\%\,\hbox {CI}=[-0.03, 0.00]$, $t_{(9287)}=-2.01$, $p=0.044$; Table S4—Model 2; Fig. 4a). In other words, more apathetic individuals showed less evidence of increased subjective utility when the magnitude of the objective reward available increased. Follow-up parcellation of this result into the individual sub-scales of the AMI questionnaire revealed that it was predominantly driven by emotional apathy (Interaction AMI-emotional $\times$ $R_0$: $\beta = -0.02$, $95\%\,\hbox {CI}=[-0.04,0.00]$, $t_{(9287)}=-2.48$, $p=0.013$; Table S5). No other interaction with experimental factors was significant for neither impulsivity nor apathy (Table S4—Model 2; Fig. 4).

## Discussion

Although it may seem counter-intuitive, a growing evidence base suggests that apathy and impulsivity can co-occur in psychiatric and neurological patients^13–16,25,28–30^ and in the general population^6,12^. The present study aimed to use behavioural tools to uncover some of the mechanisms allowing for this co-occurrence to exist. Using a new version of the TLT^38–40^, we found that although apathy and impulsivity positively correlated in self-report questionnaires, these two motivational traits were associated with unique, distinct behavioural signatures on the task. On the one hand, impulsivity was associated with an urge to respond faster, regardless of task constraints (Fig. 3). On the other hand, apathy was characterised by reduced response invigoration with higher reward (Fig. 4) as well as decoupling of reward and fatigue (the latter result is presented and discussed in *Supplementary Information*; Fig. S9). Crucially, we found no instance in which apathy significantly influenced behaviour in the opposite direction as impulsivity. That is, there was no support for the motivational spectrum hypothesis^11,17–21^.

The finding that impulsivity was associated with faster responding on the TLT is consistent with previous observations^38^, and with a broader literature that used various tasks^53,54^ (in particular go-no go or stop-signal tasks). Because it leads to larger reward on the TLT, this mode of responding had previously been assumed to capture a form of “functional” impulsivity, that is the tendency to act rapidly *when this response style is optimal*^55^. By manipulating the cost-benefit structure of the TLT ($R_0$ and $\lambda$; Fig 2b), the present experiment tested directly whether people responded to task environment. The results revealed that the TLT was indeed able to capture a behavioural manifestation of “functional” impulsivity since participants were incentivised to respond earlier when greater reward was available and when it was set to decay faster after the green onset (Fig. 3). This is in line with previous studies demonstrating a motivational role of both reward ($R_0$) and punishment ($\lambda$) in motor decision-making^56–59^.

The cost-benefit structure of our TLT was such that it was actually always financially advantageous to anticipate the green onset, regardless of the current $R_0$-$\lambda$ combination. What varied across experimental conditions was the financial benefit associated with doing do. Thus, a truly rational agent performing this new version of the TLT should have invariantly aimed to respond at green onset with as little variance as possible since this strategy maximised return. The notion of Expected Value of Control^60–62^ offers a theoretical framework for understanding why human participants may have deviated from this strategy to instead adjust their response speed to $R_0$ and $\lambda$. According to this framework, exerting control (to generate the response that harvests most reward) comes at a cost that is weighted against expected reward to determine the optimal amount of control to invest in the ongoing task. If accurately anticipating the green onset comes at an extra cost—potentially due to more precise temporal estimation and greater attentional resources—then it may become optimal to invest these additional resources only when the associated pay-off is high enough (large reward available and/or large cost of generating late responses). Within the two-horse linear rise-to-threshold model^38–40^ developed in *Supplementary Information* (Figs. S4–S5), $R_0$ and $\lambda$ were indeed found to increase the accumulation rate of the anticipatory decision process, thereby allowing the model to generate responses that fell closer to the green onset and were less variable (Fig. S8). Our results are therefore consistent with the idea that reward ($R_0$) and punishment ($\lambda$) exerted their motivational influence on the TLT through the optimisation of the anticipatory process, which in itself may have come at an intrinsic cost. Apathy and impulsivity were not found to interact with this process in the current study.

Although all individuals strategically tailored their response speed to the cost-benefit structure, the psychometric trait measure of impulsivity derived from the BIS-11 and UPPS-P questionnaires was instead related to a rigid readiness to respond earlier on the TLT, irrespective of experimental condition (Fig. 3). This can be seen as a form of “dysfunctional” impulsivity which incidentally happened to be beneficial on the TLT. Within the modeling framework developed in *Supplementary Information*, this behaviour was conceptualised as a bias towards engaging the anticipatory process in the task at baseline prior to any exposure. Consistent with this idea, the greatest contributor of the relationship between impulsivity score and RT on the TLT was “Positive Urgency”, a subscale of the UPPS-P that has previously been linked to a difficulty inhibiting prepotent motor responses^63,64^. We therefore propose that inhibitory control deficit may be a dimension of impulsivity that sits outside the dopamine-mediated motivational spectrum. Inhibitory control deficits have indeed been reported in both impulsive and apathetic patients with a rather specific neuroanatomical^13,30,65,66^ (ventral prefrontal cortex) and neurochemical^67–70^ (serotoninergic and noradrenergic deficiency) signature that contrasts with the neurophysiological basis of the motivational spectrum hypothesis (dopaminergic frontostriatal circuits^11,22–26^). This anatomical segregation may account for the possible co-occurrence of inhibitory control deficits with other dimensions of apathy and impulsivity.

The use of a hand-held dynamometer allowed us to measure response vigour in addition to speed in our version of the TLT. As expected^52^, this offered an indirect read out of *wanting*^71^ as participants were found to squeeze harder when the reward available increased (Fig. 4). It is worth noting the absence of effect of decay rate ($\lambda$) on vigour, suggesting this behavioural metric was related to the reward available presented on the screen ($R_0$) as opposed to expected outcome. Reward sensitivity is one of the pillar candidate mechanism of the motivational spectrum hypothesis^11^ since it involves dopaminergic frontostriatal circuits that can be up- or down- regulated in disease and pharmacologically, and it shares conceptual links to both apathy and impulsivity depending on the direction of the impairment (too little: apathy; too much: impulsivity).

Although the role of altered reward sensitivity in the genesis of apathy is well established^42–44,72–76^, its contribution to impulsivity is more debated, with many authors arguing for a distinction between these two concepts – so called “reward (hyper)sensitivity” vs. “rash impulsiveness”^77–79^. Within the impulsivity literature, “reward (hyper)sensitivity” refers to a purposeful drive to obtain rewarding stimuli, while “rash impulsiveness” is defined as the tendency to act spontaneously with disregard for potential consequences. The psychometric measure of impulsivity used in our study seemed to be more specific to rash impulsiveness given its relationship to RT on the TLT (Fig. 3). On the other hand, the apathy score derived from the AES and AMI questionnaires was associated with blunted reward sensitivity on the TLT, i.e., lower invigoration of the manual response by monetary incentives. Thus, apathy seems to be more specifically a disorder of the valuation system, compared to impulsivity. Note that this finding does not invalidate the existence of a potential motivational spectrum for reward sensitivity. For example, it might be the case that reward hypersensitivity only leads to impulsive behaviour in the presence of a conjoint inhibitory control deficit^79^.

Overall, the findings presented here suggest that impulsivity may have a direct effect on action initiation/inhibition, whereas apathy appears to be more specifically related to the valuation system. These distinct mechanisms may contribute to the possible co-expression of both apathy and impulsivity in healthy individuals. From a methodological perspective, our results show the utility of our modified TLT for studying these two motivational traits conjointly within a single controlled experimental setting. Future studies may consider applying this approach to clinical populations in which apathy and impulsivity are known to co-occur.

## Methods

### Participants

Sixty young healthy adults (30 females, mean age: 24.97 years, *s.d.*: 4.85) took part in the study. They were recruited via the Oxford Psychology Research participant recruitment scheme website (https://opr.sona-systems.com) and online study advertisement. Volunteers who reported a personal history of neurological or psychiatric condition or undertaking psycho-active medication were excluded from participation. The study was carried out in accordance with the Declaration of Helsinki and with the ethical approval from the University of Oxford Central University Research Ethics Committee. All participants gave written informed consent before inclusion. Data collection took place in person between October 2019 and February 2020.

### External measures

Participants completed two well-established self-report questionnaire measures of apathy (Apathy Evaluation Scale, AES^7^; Apathy Motivation Index^10^) and impulsivity (Barratt Impulsiveness Scale, BIS-11^8^; Urgency, Premeditation (lack of), Perseverance (lack of), Sensation Seeking, Positive Urgency, Impulsive Behavior Scale, UPPS-P^9^). The use of two questionnaires per construct provided a more reliable measure of apathy and impulsivity (see Fig. 1 for cross-questionnaire correlations).

Details about the four questionnaires are provided below:

- *Apathy Evaluation Scale*^7^ (AES): 18-item questionnaire scored on a 4-point Likert scale (total scores ranging from 18 to 72), subdivided into 4 sub-scales (emotional apathy, behavioural apathy, cognitive apahty, other);
- *Apathy Motivation Index*^10^ (AMI): 18-item questionnaire scored on a 5-point Likert scale (total scores ranging from 0 to 4), sub-divided into 3 sub-scales (emotional apathy, behavioural apathy, social apathy);
- *Urgency, Premeditation (lack of), Perseverance (lack of), Sensation Seeking, Positive Urgency, Impulsive Behavior Scale*^9^ (UPPS-P): 59-item questionnaire scored on a 4-point Likert scale (total scores ranging from 59 to 236), sub-divided into the 5 sub-scales (negative urgency, lack of premeditation, lack of perseverance, sensation seeking, positive urgency);
- *Barratt Impulsiveness Scale*^8^ (BIS-11): 30-item questionnaire, scored on a 4-point Likert scale (total scores ranging from 30 to 120), sub-divided into 3 sub-scales (attentional impulsiveness, motor impulsiveness, non-planning impulsiveness).

### Apparatus

Behavioural testing took place in a quiet room with only the participant and the experimenter present. All tasks were presented on a 17-inch touchscreen PC using MATLAB version 2018 (MathWorks; https://uk.mathworks.com) and Psychtoolbox^80^ (version 3). Force responses were recorded using a dynamometer (SS25LA, BIOPAC Systems) held in the dominant hand.

### Maximum voluntary contraction (MVC)

At the beginning of the experimental session, participants were asked to squeeze the hand-held dynamometer as hard as they possibly could on three consecutive occasions (5 seconds per squeeze). The strongest force recorded during this procedure was defined as the Maximum Voluntary Contraction (MVC), and used for task calibration purposes.

### Modified traffic light task

The Traffic Light Task (TLT) is a well-established test of rapid, opportunistic decision-making under temporal uncertainty in pursuit of risky, time-sensitive reward^38–40^. The current version of the task departed from the one used in previous studies in two important ways (Fig. 2). First, a hand-held dynamometer was used to record the response, as opposed to an eye movement^39,40^ or a button press^38^. This provided a measure of vigour as an indirect index of subjective utility^52^, as well as introduced a fatigue-inducing physical effort dimension to the task. Second, both the starting point ($R_0$) and decay constant ($\lambda$) of the reward function (Eq. 2) were manipulated in a two-by-two design in order to investigate the influence of cost-benefit structure on performance.

At the beginning of each trial, participants were presented with a picture of a coin (20p or $\pounds$1) and an animal (turtle or hare) that informed them of the cost-benefit features of the trial ($R_0$-$\lambda$ combination). They subsequently had to press the space bar with their non-dominant hand to self-initiate each trial. Upon release of the space key, the screen background turned red, then amber, then green. The red and green colours both stayed on the screen for 800 ms. The duration of the amber light, however, could take one of three values. It was 1000 ms in most cases (69.2% of all trials, i.e., 72 trials per condition in total), but departed from this time by ± 300 ms in the remaining cases (30.8% of all trials., i.e., 16 trials per condition and per deviation in total). Experimental blocks included 13 repetitions of each of the four possible $R_0$-$\lambda$ combinations (52 trials per block in total). Nine of these trials had the most common amber light duration (1000 ms), while two had a shorter one (700 ms), and two had a longer one (1300 ms). The order of trials was random such that rarer amber light durations occurred unexpectedly. Note, however, that rare trials (700 ms or 1300 ms amber duration) were always followed by a common trial (1000 ms amber light duration).

The task statistics allowed participants to form an internal representation of the most common amber light duration (see *Predictive control task*), and use this knowledge to anticipate the green onset if they wished. Thus, on a trial-by-trial basis, people had to rapidly decide whether to wait for the green light to appear before generating a (reactive) response, or whether to respond based on their knowledge of task statistics but risk incurring a penalty (anticipatory response).

Participants were instructed that they could win hypothetical money by “catching the animal” as quickly as they could once the screen turned green. To catch the animal, they had to squeeze the hand-held dynamometer with a force of at least 15% of their MVC. They were explicitly informed that stronger squeezes did not yield higher reward. A vertical progress bar provided visual feedback on the current (sub-threshold) force exerted. Upon crossing the response force threshold (15% MVC), an outline appeared around the picture of the animal to indicate that the response had been recorded. It was red if the response occurred before the green onset (penalised anticipatory response), and black otherwise (rewarded response). The trial outcome was subsequently presented for 2 seconds. Reward was calculated according to the following inverse exponential temporal discount function (Fig. 2c):

2 $$\begin{aligned} {\left\{ \begin{array}{ll} &{}R(RT)\ =\ R_0 \times e^{-\lambda \times RT} \ \ \ \text {if} \ RT\ge 0\\ &{}R(RT)\ = -0.05 \ \ \ \text {if} \ RT<0 \end{array}\right. } \end{aligned}$$

where $R_0$ refers to the magnitude of the reward available ($\pounds$1 or $\pounds$0.20), $\lambda$ refers to the rate of its decay after green onset ($\lambda _{fast}=0.0125$ $\pounds$.ms$^{-1}$ and $\lambda _{slow} = 0.0033$ $\pounds$.ms$^{-1}$, which correspond to a half-life of 55 ms and 208 ms, respectively), and RT refers to the time elapsed between the green onset and the crossing of the 15%MVC threshold. The outcome of each trial was added to the amount in the “bank”, displayed at the top of the screen. In-between blocks, a score board showed to amount earned in each block, ranked from highest to lowest. To keep participants motivated, they were encouraged to beat their own score from one block to the next. The amount in the bank was reset to zero at the beginning of each task block.

Participants underwent eight blocks of 52 trials each (mean block duration: 4.87 min, *s.d.*: 0.34 min). To limit the cognitive load, the reward decay rate (animal) was held constant for the first and second half of each block, and changed only once, half-way through the block (after 26 trials). It alternated in a “AB-BA” fashion over a two-block period with half of the participants (15 females, 15 males) starting with the quick decay condition (FS-SF-FS-SF-FS-SF-FS-SF), and the other half starting with the slow decay condition (SF-FS-SF-FS-SF-FS-SF-FS). The amount of reward available ($\pounds$1 or $\pounds$0.20), by contrast, varied randomly on a trial-by-trial basis. All blocks included the same number of each trial type although in different order.

Control tasks for (1) simple RT, (2) anticipatory abilities, and (3) time discrimination are described and analysed in *Supplementary Information*.

### Fatigue ratings

Because of the physical effort component of this version of the TLT, participants were asked to rate their level of subjective fatigue throughout the experimental session. When doing so, a vertical visual analogue scale (VAS) appeared in the middle of the screen with the words “Not at all” and “Extremely” displayed at its lowest and highest extremes, and the question “How TIRED do you feel?” at the top (Fig. S2d). Individuals then placed a cursor at the level that best reflected their current fatigue by touching the screen, and pressed the space bar to validate their rating. The percentage corresponding to the level selected was displayed on the side of the cursor. Within a task, the initial position of the cursor was set to the value of the preceding rating.

The reactive control task included four fatigue ratings in total (one every 40 trials), the TLT task four fatigue ratings per block (one every 13 trials), and the temporal duration discrimination task four fatigue ratings in total (one every 16 trials). Analysis and discussion of fatigue data is provided in *Supplementary Information*.

### Statistical analysis

Data handling and statistical analysis were done in MATLAB (The Math-Works Inc., version 2020a). On the TLT, were excluded from the analysis trials in which: (1) no response was recorded ($0.88\%$ of the data); (2) a response was recorded earlier than 1000 ms before the green onset ($0.10\%$ of the data); (3) a response was recorded later than 1000 ms after the green onset ($0.10\%$ of the data). The behavioural measure of interest were: (1) Earnings (i.e. the amount of reward obtained); (2) Anticipation (i.e. the percentage of trials in which participants responded before the green onset); (3) Reaction Time (RT) relative to the green onset; (4) Vigour (i.e. peak force exerted within a trial, expressed as a percentage of the MVC). Peak acceleration of the response movement showed strong covariance with vigour ($R^2 = 0.69$) and was therefore not treated as a behavioural measure of interest as it carried little unique variance.

Statistical analysis used general linear mixed-effect models (LMM). In order to ensure generalizability of our results, LMMs included the maximal random effects structure allowed by the design^81^. That is, explanatory variables that contained multiple values at the individual level (Experimentally manipulated variables: $R_0$, $\lambda$, block index, $a_{n-1}$, $a_{n}$; Task measures: vigour, RT, block-averaged earnings, fatigue ratings) were entered with subject- and gender- specific random slopes. Variables that contained only one measure per individual (e.g., questionnaire-derived scores, total earnings, total anticipation, age) included a gender-specific random slope. All models also included a random intercept at the individual and gender level. Note that gender was treated as a random effect in the present study because we did not intend to investigate its effect on task performance or questionnaire responses. This approach ensured that our results generalized across gender categories and that they were not driven by eventual systematic gender-related differences. When analysing the relationship between apathy and a behavioural variable, the other motivation trait (impulsivity) was systematically included in the model (partial correlation). Similarly, when analysing the relationship between impulsivity and a behavioural variable, the model systematically controlled for apathy. All explanatory variables were z-scored before entering the LMMs to obtain standardised parameter estimates. All statistical tests were two-sided. All LMM equations are provided in *Supplementary Information*.

## Supplementary Information

Supplementary Information.

## Data Availability

Anonymised data and code for replicating the main results in the manuscript have been deposited on the Open Science Framework platform: https://osf.io/6y54r/?view_only=c4d38174393d4568948e6287f498db88.
