# Supplementary material for: Dissociable behavioural signatures of co-existing impulsivity and apathy in decision-making
Source: Sci Rep. 2022 Dec 12;12:21476. doi: 10.1038/s41598-022-25882-z (PMC9744918; doi:10.1038/s41598-022-25882-z)
Supplement: Supplementary file 1 — Supplementary Information. [file 41598_2022_25882_MOESM1_ESM.pdf]

Supplementary Information

Dissociable behavioural signatures of co-existing impulsivity and apathy in decision-making

Pierre Petitet, Sijia Zhao, Daniel Drew, Sanjay G. Manohar, and Masud Husain

Supplementary Methods

Reactive Control Task

Before undergoing the TLT, participants performed a short control task (mean duration: 5.77 min, *s.d.*: 0.49) aimed at measuring their responsiveness to unpredictable changes in screen background colour (Fig. S2a). A secondary objective of this task was to familiarise participants with the visual display and mode of responding of the TLT. The background was grey and turned green for 800 ms every 800 to 2800 ms according to a pseudo-uniform distribution. Participants were instructed to squeeze the hand-held dynamometer as quickly as they could in response to the green light. When reaching 15% of the MVC, the progress bar filled up, and a black outline appeared around a grey rectangle indicating that a response had been recorded. This short control task included three blocks of 40 trials each, separated by a fatigue rating.

Predictive Control Task

Anticipatory behaviour on the TLT may in part reflect statistical learning. That is, in order to respond at green onset, individuals need to build an accurate internal representation of the distribution of amber light durations. In order to track how this ability developed over the course of the TLT, a predictive response task was interleaved at the end of each TLT block (Fig. S2b). In this short task (10 trials per block; mean block duration: 1.13 min, *s.d.*: 0.22), the background turned red for 800 ms, then amber, but did not turn green. Instead, participants were instructed to respond when they thought the background should turn green (i.e., after 1000 ms on average). On each trial, after the response was recorded, a visual representation of the most frequent timeline (red/amber/green: 800/1000/800 ms) was presented on the screen and participants had to report whether they thought they had responded too early, on time, or too late by positioning an arrow at the time of their response. This rating provided a measure of metacognitive accuracy, i.e., the precision of ones' perception of their own performance. No performance feedback was provided on the predictive control task.

Temporal Duration Discrimination Task

In order to investigate the extent to which performance on the TLT was influenced by inter-individual differences in time perception, participants performed a two-alternative forced choice temporal duration discrimination task at the end of the experimental session (Fig. S2c; 64 trials; mean duration: 7.38 min, *s.d.*: 0.51). The task was adapted from Bueti et al. (2008)[1], task B, and was similar to the one used by Burnett et al. (2012)[2]. The screen turned amber twice in a row with a grey interval of 800 ms in-between. On each trial, one of the two amber durations was 1000 ms (the "reference" duration), equivalent the mean and most frequent duration on the TLT (Fig. S2a). The other amber duration (the "test" duration) was either 700 ms (short amber light duration on the TLT), 850 ms, 1150 ms or 1300 ms (long amber light duration on the TLT). The order of reference and test durations was fully randomised across trials (16 repetitions per test duration). Participants pressed the left or right arrow keys with the index and middle fingers of their dominant hand to indicate whether the first or second flash lasted longer. On half of the trials, the left arrow key corresponded to the first flash, while on the other half it corresponded to the second flash. The key-response matching was explicitly displayed on the screen, and reversed only once halfway through the task. No performance feedback was provided on the temporal duration discrimination task.

Mixed-effect model equations

$$\begin{aligned} Total\ earnings &\sim 1 + Percentage\ Anticipation + Vigour \\ &+ (1 + Percentage\ Anticipation + Vigour \mid Gender) \end{aligned} \tag{S1}$$

$$\begin{aligned} Trial\ earning &\sim 1 + RT + Vigour + R_0 + \lambda + a_n \\ &+ (1 + RT + Vigour + R_0 + \lambda + a_n \mid Block) \quad + (1 + RT + Vigour + R_0 + \lambda + a_n \mid Participant) \end{aligned} \tag{S2}$$

$$Trial\ RT \sim 1 + Vigour + (1 + Vigour \mid Participant) \tag{S3}$$

$$\begin{aligned} Total\ earnings &\sim 1 + Apathy\ Score + Impulsivity\ Score + Age \\ &+ (1 + Apathy\ Score + Impulsivity\ Score + Age \mid Gender) \end{aligned} \tag{S4}$$

$$\begin{aligned} Mean\ RT \sim\ & 1 + a + a_{n-1} + ln(Block) + R_0 + \lambda + R_0 : \lambda \\ & + (1 + a + a_{n-1} + ln(Block) + R_0 + \lambda + R_0 : \lambda \mid Participant) \end{aligned} \tag{S5}$$

$$\begin{aligned} Mean\ RT \sim\ & 1 + a + a_{n-1} + ln(Block) + R_0 + \lambda \\ & + Imp + a : Imp + a_{n-1} : Imp + ln(Block) : Imp + R_0 : Imp + \lambda : Imp \\ & + Ap + a : Ap + a_{n-1} : Ap + ln(Block) : Ap + R_0 : Ap + \lambda : Ap + Age \\ & + (1 + a + a_{n-1} + ln(Block) + R_0 + \lambda \\ & + Imp + a : Imp + a_{n-1} : Imp + ln(Block) : Imp + R_0 : Imp + \lambda : Imp \\ & + Ap + a : Ap + a_{n-1} : Ap + ln(Block) : Ap + R_0 : Ap + \lambda : Ap + Age \mid Gender) \\ & + (1 + a + a_{n-1} + ln(Block) + R_0 + \lambda + R_0 : \lambda \mid Participant) \end{aligned} \tag{S6}$$

$$\begin{aligned} Mean\ Vigour \sim\ & 1 + a + a_{n-1} + ln(Block) + R_0 + \lambda + R_0 : \lambda + Mean\ RT \\ & + (1 + a + a_{n-1} + ln(Block) + R_0 + \lambda + R_0 : \lambda + Mean\ RT \mid Participant) \end{aligned} \tag{S7}$$

$$\begin{aligned} Mean\ Vigour \sim\ & 1 + a + a_{n-1} + ln(Block) + R_0 + \lambda + Mean\ RT \\ & + Imp + a : Imp + a_{n-1} : Imp + ln(Block) : Imp + R_0 : Imp + \lambda : Imp \\ & + Ap + a : Ap + a_{n-1} : Ap + ln(Block) : Ap + R_0 : Ap + \lambda : Ap + Age \\ & + (1 + a + a_{n-1} + ln(Block) + R_0 + \lambda + Mean\ RT \\ & + Imp + a : Imp + a_{n-1} : Imp + ln(Block) : Imp + R_0 : Imp + \lambda : Imp \\ & + Ap + a : Ap + a_{n-1} : Ap + ln(Block) : Ap + R_0 : Ap + \lambda : Ap + Age \mid Gender) \\ & + (1 + a + a_{n-1} + ln(Block) + R_0 + \lambda + R_0 : \lambda + Mean\ RT \mid Participant) \end{aligned} \tag{S8}$$

For the following two analyses, fatigue ratings, vigour and reward obtained were block-averaged. Time-on-task corresponded to the cumulative time reached at the end of each of the eight experimental blocks (starting from zero at the beginning of the first block). Baseline fatigue was the average fatigue rating before the start of the TLT.

$$\begin{aligned} Mean\ fatigue\ rating \sim\ & 1 + Baseline\ fatigue + Time\text{-}on\text{-}task + Vigour + Reward \\ & + (1 + Baseline\ fatigue + Time\text{-}on\text{-}task + Vigour + Reward \mid Participant) \end{aligned} \tag{S9}$$

$$\begin{aligned} Mean\ fatigue\ rating \sim\ & 1 + Baseline\ fatigue + Time\text{-}on\text{-}task + Vigour + Reward + Age \\ & + Ap + Time\text{-}on\text{-}task : Ap + Vigour : Ap + Reward : Ap \\ & + Imp + Time\text{-}on\text{-}task : Imp + Vigour : Imp + Reward : Imp \\ & + (1 + Baseline\ fatigue + Time\text{-}on\text{-}task + Vigour + Reward + Age \\ & + Ap + Time\text{-}on\text{-}task : Ap + Vigour : Ap + Reward : Ap \\ & + Imp + Time\text{-}on\text{-}task : Imp + Vigour : Imp + Reward : Imp \mid Gender) \\ & + (1 + Time\text{-}on\text{-}task + Vigour + Reward \mid Participant) \end{aligned} \tag{S10}$$

Computational model of reaction time distributions

On the TLT, the distribution of reaction times (RT) relative to the green onset is characterised by the presence of two distinct peaks – corresponding to *anticipatory/predictive* and *reactive* responses – whose relative magnitude is influenced by the duration of the amber light[2–4] (Figs. S6-S8). Trials in which the amber light lasts longer than usual typically lead to more anticipatory (early) responses, while trials in which the amber light is shorter are characterised by predominantly reactive (late) responses. This data feature is well reproduced by a two-horse linear rise-to-threshold model in which processes compete (or "race") towards a threshold in order to generate a response[2–4]. According to this two-horse linear rise-to-threshold model, the

probability that a response has occurred by time t following amber light onset (i.e., the cumulative probability distribution) is given by:

$$Pr(T \leq t|n) = \Psi(t, \mu_A, \sigma_A^2) + \Psi(t - a_n, \mu_R, \sigma_R^2) - \Psi(t, \mu_A, \sigma_A^2) \times \Psi(t - a_n, \mu_R, \sigma_R^2) \tag{S11}$$

where Ψ is the cumulative recinormal distributions[5], and a_n is the amber light duration of the n-th trial (i.e., either 700, 1000, or 1300 ms). The cumulative recinormal distribution is defined for $t > 0$ as:

$$\Psi(t, \mu, \sigma^2) = 1 - \Phi\left(\frac{1/t - \mu}{\sigma}\right) \tag{S12}$$

where μ and σ^2 are the mean and variance of the standard cumulative normal distribution Φ. Thus, within the two-horse linear rise-to-threshold modeling framework, each process (anticipatory and reactive) is parameterised in terms of the mean and variance of its rate-of-rise. The anticipatory process is assumed to start rising (with mean rate-of-rise μ_A and variance σ_A^2) from amber onset onwards, thereby crossing the response threshold around the average green onset. The reactive process, on the other hand, is automatically triggered by the actual onset of the green light, and rises more sharply (with mean rate-of-rise μ_R and variance σ_R^2).

By design, this formulation (Eq. S11) is solely capable of capturing how the duration of the amber light of a given trial (a_n) impacts the relative contribution of the anticipatory and reactive processes to the RT distribution. However, a simple analysis of mean reaction times using a LMM demonstrated that at least three other factors also had an impact on the average speed of the response (Fig. 3), namely:

- Amber light duration of the *previous* trial, a_{n-1};
- Task practice, indexed by the trial number, n;
- The cost-benefit structure of the trial (R_0 and τ).

The goal of incorporating these effects within a computational model (two-horse model) was to gain further mechanistic insight by considering the full RT distribution (as opposed to mere mean RT). In other words, the question asked was: what is the putative mechanism by which any of these experimental factors influence reaction time? For simplification purposes, the two experimental variables defining the cost-benefit structure of the trial (R_0 & λ) were assumed to both affect the same compartment of the model. For each factor (a_{n-1}, n, R_0 & λ), we tested three candidate computational mechanisms by which the factor could exert its influence (Fig. S4):

- By advancing/postponing the time at which the anticipatory process starts rising towards the response threshold, t_0 (Fig. S4b);
- By increasing/decreasing the mean rise of the anticipatory process, μ_A (Fig. S4c);
- By increasing/decreasing the probability of the anticipatory process to be active on a given trial, w_n (Fig. S4d).

Incorporating these candidate mechanisms into the two-horse linear rise-to-threshold model required extending Eq. S11 as follows:

$$\begin{aligned} Pr(T \leq t|n) =& w(n) \times [\Psi(t - \delta(n), \mu_A(n), \sigma_A^2) + \Psi(t - a_n, \mu_R, \sigma_R^2) - \Psi(t - \delta(n), \mu_A(n), \sigma_A^2) \times \Psi(t - a_n, \mu_R, \sigma_R^2)] \\ &+ (1 - w(n)) \times \Psi(t - a_n, \mu_R, \sigma_R^2) \end{aligned} \tag{S13}$$

where $w(n)$ is the probability of the anticipatory process to be active on the n-th trial ($0 \leq w(n) \leq 1$), $\delta(n)$ is the shift in starting time of the anticipatory process relative to the amber onset on the n-th trial, and $\mu_A(n)$ is the mean rate-of-rise of the anticipatory process on the n-th trial.

As illustrated in Figure S4, each of these alternative mechanisms (manipulating $\delta(n)$, $\mu_A(n)$ or $w(n)$) has a unique, distinct, signature on the RT distribution. This mathematical property made it possible to determine, for each experimental factor (a_{n-1}, n, [R_0 & λ]), the candidate computational mechanism ($\delta(n)$, $\mu_A(n)$ or $w(n)$) that best explained its impact on human performance.

$\delta(n)$ and $\mu_A(n)$ were assumed to be a linear combination of the experimental factors influencing them, that is:

$$\begin{aligned}\delta(n) &= X\beta \\ \delta(n) &= X\beta\end{aligned} \tag{S14}$$

where X is in a design matrix containing the experimental factors of interest and β is a vector of parameter estimates capturing the magnitude of these effects. Because $w(n)$ was bounded between 0 and 1, it was assumed to have instead a logistic relationship to the experimental factors of interest, that is:

$$w(n) = \frac{1}{1 + e^{-X\beta}} \tag{S15}$$

Note that the experimental factors included in a given design matrix were z-scored at the individual level to facilitate model fitting.

By considering all possible combinations of experimental factor (a_{n-1}, n, [R_0 & λ]) *vs.* computational mechanism ($\delta(n)$, $\mu_A(n)$ or $w(n)$), twenty-seven models were created ($3^3 = 27$ combinations; Fig. S5a). In addition to these 27 models, we also tested the original two-horse linear-to-arise model described in Equation S11[3] (Reference model 1) as well as its simple upgrade (Reference model 2) that included a subject-specific probability of anticipating w that was fixed across trials.

Supplementary Results

Control tasks

Reactive Control Task

The Reactive Control Task was used to measure participants' ability to respond to unpredictable changes in screen background colour. Since no anticipation was possible, RT was thought to primarily reflect delay due to visuomotor processing. To investigate the relationship of apathy and impulsivity with this process, a LMM was built with the two questionnaire-derived scores as predictors of mean RT, controlling for age (fixed effect) and gender (random effect). Trials for which a response occurred within the 800 ms preceding a change in background colour were excluded from the analysis (0.67% of the data excluded).

Mean RT on the Reactive Control Task showed no relationship to neither apathy ($\beta = 0.00$, $95\%CI = [-0.01, 0.01]$, $t_{(56)} = 0.30$, $p = 0.77$; Table S8) nor impulsivity ($\beta = 0.01$, $95\%CI = [-0.01, 0.02]$, $t_{(56)} = 0.89$, $p = 0.38$; Table S8). This null result reinforces the idea that faster responses of more impulsive individuals on the TLT is due not to shorter visuomotor delays but rather to a greater tendency to anticipate.

Predictive Control Task

At the end of every TLT block, participants underwent a short Predictive Control Task in which they were explicitly instructed to respond based on their internal estimate of the green onset (see *Supplementary Methods*). The reaction time (RT) was expressed in relation to the most common green onset (i.e., 1000 ms after the amber onset). Trials for which the RT was further than three scaled median absolute deviations (MAD) away from the median of the participant were excluded from the analysis (5.27% of the data excluded).

The variation of average RT over successive blocks was used as a measure of predictive learning. A linear mixed-effect model (LMM) showed that the average RT improved over successive blocks (Main effect of log-transformed Block on block-averaged RT: $\beta = -0.10$, $95\%CI = [-0.16, -0.03]$, $t_{(478)} = -2.87$, $p = 0.0042$; Table S9 – Model 1; Fig. S3). Note that this constitutes a much less pronounced reduction in RT compared to the one observed in the TLT ($\beta = -0.23$, $95\%CI = [-0.27, -0.19]$; Table S2). Follow-up pairwise t-test comparisons of block averaged RTs showed that performance reached an asymptotic level as early as the second block since only the first block significantly differed from all others ($p < 0.001$, not Bonferroni-corrected). Furthermore, participants responded earlier on average on the Predictive Control Task compared to the TLT in the first and second blocks (paired t-tests: both $p < 0.01$).

Performance on the Predictive Control Task therefore indicates that, when instructed to do so, participants were well able to anticipate the green light onset. Nevertheless, the strategy adopted by participants on the TLT task led to slower mean RT, especially in the first two experimental blocks (Fig. S3a). This is consistent with the idea that the learning effect observed

on the TLT only marginally reflects predictive learning but instead seem to derive from a shift in strategy from reaction to anticipation.

To investigate the relationship of apathy and impulsivity with predictive learning, the two questionnaire-derived scores were added to the LMM while controlling for age (fixed effect) and gender (random effect). Although impulsivity did not significantly interact with predictive learning (Interaction Impulsivity score × log-transformed Block on block-averaged RT: $\beta = 0.02$, $95\%CI = [-0.06, 0.11]$, $t_{(472)} = 0.55$, $p = 0.58$; Table S9 – Model 2), apathy did (Interaction Apathy score × log-transformed Block on block-averaged RT: $\beta = -0.12$, $95\%CI = [-0.18, -0.06]$, $t_{(472)} = -3.82$, $p < 0.001$; Table S9 – Model 2). That is, greater Apathy scores were associated with faster reduction of mean RT over successive blocks in the Predictive Control Task.

After squeezing the dynamometer, participants indicated when they thought they responded in comparison to when they should have responded. The position of the cursor they placed allowed us to calculate an "estimated RT" in relation to the theoretical green onset. The relationship between the estimated and actual RT gave us an measure of metacognitive abilities, i.e., how well individuals could perceive/report their own performance.

On average, there was a good agreement between actual and estimated RT (both RT and estimated RT z-scored within-individuals; slope: $\beta = 0.49$, $95\%CI = [0.38, 0.52]$, $t_{(4545)} = 12.41$, $p < 0.001$), indicating accurate metacognition. Adding the two questionnaire-derived scores to the LMM while controlling for age (fixed effect) and gender (random effect) showed no interaction of these traits with metacognition (both $p > 0.19$).

Temporal Duration Discrimination Task

The Temporal Duration Discrimination Task was used to measure inter-individual differences in time perception. The effect of test duration on the response was analysed using the following logistic mixed-effect model:

$$Responded\ \text{"}Test\ duration\ is\ longer\text{"} \sim 1 + Test\ duration + (1 + Test\ duration \mid Participant) \qquad \text{(S16)}$$

Overall, participants' responses were accurate (slope: $\beta = 1.64$, $95\%CI = [1.47, 1.80]$, $t_{(3838)} = 19.23$, $p < 0.001$; Table S10 – Model 1). Adding questionnaire-derived scores to the logistic regression showed no significant interaction of apathy and impulsivity with the accuracy of time discrimination (Apathy score × accuracy: $\beta = -0.07$, $95\%CI = [-0.21, 0.07]$, $t_{(3834)} = -0.95$, $p = 0.34$; Impulsivity score × accuracy: $\beta = 0.00$, $95\%CI = [-0.13, 0.14]$, $t_{(3834)} = 0.07$, $p = 0.94$; Table S10 – Model 2).

A computational model of RT

The goal of this supplementary analysis of TLT data was to take advantage of the greater information carried by the full distribution of RT to characterise the impact of impulsivity on the putative cognitive mechanisms involved. It is well established that RT on the TLT follows a bimodal (double recinormal) distribution with an early "anticipatory" and a late "reactive" peak whose relative magnitude depends on the duration of the amber light[2–4]. This data feature is well reproduced by a two-horse linear rise-to-threshold model, which posits the existence of two competing decision processes – an anticipatory and a reactive one – racing towards a fixed response threshold. The anticipatory process is thought to capture an urge to respond that starts building up from amber onset onwards and culminates at the green onset. This can be seen as a direct manifestation of functional impulsivity on the TLT. By contrast, the reactive process captures to the response to the onset of the green light.

Visual inspection of the RT distribution in our modified TLT confirmed the presence of distinct anticipatory and reactive peaks, which were more or less ample depending on the duration of the amber light (Figs. S6-S8). When the amber light duration was shorter than usual, the RT distribution was dominated by the late reactive peak, whereas when the amber light lasted longer than usual, the RT distribution was dominated by the early anticipatory peak. Crucially, the other experimental factors considered in the main text (a_{n-1}, block index, R_0, λ) appeared to have distinctive signatures on the RT distribution. In order to capture such effects, we extended the two-horse linear rise-to-threshold model to allow for three candidate mechanisms by which the factors may influence the model (see *Supplementary Methods*):

- by shifting the time at which the anticipatory process starts rising, t_0 (Fig.S4b);
- by changing in the rate at which the anticipatory process rises toward the response threshold, μ_A (Fig.S4c);
- by changing the probability of the anticipatory process to be active on a given trial, w (Fig.S4d)

As illustrated in Fig.S4b-d, each of these three candidate computational mechanisms was associated with a unique, distinct signature on the RT distribution, making it possible, through model fitting and comparison, to determine the mechanism that best explained the effect of each experimental factor. Variants of the two-horse linear rise-to-threshold model were created by testing potential candidate mechanisms for each experimental factor. To reduce the number of models to test, cost-benefit parameters (R_0 & λ) were assumed to operate through the same computational mechanism (see *Supplementary Methods*).

All combinations of three potential mechanisms (effect on t_0, μ_A, w) to explain three experimental factors (a_{n-1}, trial index, cost-benefit structure) led to 27 (i.e., 3^3) candidate models. Two additional models were tested for reference. They did not include a sensitivity to any of the three experimental factors, and merely differed in terms of their architecture: (1) two-horse model with an anticipatory process that was always active (i.e., $w = 1$; Reference model 1, equivalent to the model used in previous studies[2–4]); (2) a "weighted" version of the same model in which the anticipatory process had a fixed probability w ($0 \le w \le 1$) to be activated. Models were fitted to individual datasets separately, using a maximum likelihood procedure with a gradient-based method *fminsearch* implemented in MATLAB (The MathWorks inc., version 2020a). Model comparison was carried out with Bayesian Information Criterion (BIC), summed across all individual datasets. This model selection method was chosen for its stringent penalisation of model complexity.

The introduction of a probabilistic activation of the anticipatory process induced a large explanatory gain compared to the original two-horse model (Reference model 2 vs. Reference model 1: $\Delta BIC = -1,117.03$; Fig. S5a). Further, compared to simpler, context-insensitive reference models, incorporating a sensitivity to task statistics, practice and cost-benefit structure provided a better account of the data (lower BIC in Models 1-27 relative to Reference models 1-2; Fig. S5a). Overall, the winning model (lowest BIC score; Model 4 vs. Reference Model 1: $\Delta BIC = -7,076.87$; Model 4 vs. Model 17: $\Delta BIC = -213.74$; Fig. S5a) accounted for these effects in the following ways:

- The amber duration of the previous trial influences the mean rate-of-rise of the anticipatory process (faster/slower μ_A when the previous amber light was shorter/longer than usual);
- The probability of the anticipatory process to be active (w) grows logarithmically with task practice (trial index);
- The cost-benefit structure of the trial influences mean rate-of-rise of the anticipatory process (faster/slower μ_A when more/less reward is available and when the reward decays faster/shorter after the green onset).

Fig. S5b provides a visual illustration of the best model's architecture, and Figs. S6 to S8 show model fit of the pooled group RT distributions. Note that, in this model, learning is parameterised as an increase in the probability of the anticipatory process to be active (purple curve in Fig. S5b). In that respect, individuals can differ in term of the baseline w (intercept), as well as the speed at which w rises across successive trials.

In order to determine which features of the model conferred a functional advantage on the TLT, we investigated the relationship between parameter estimates and total earnings. These correlations were run separately for each of the nine model parameters with Spearman correlation after outlier removal (exclusion of parameter estimates further than three scaled median absolute deviations away from the median of the group), and controlled for Age and Gender (partial correlation). The mean rate-of-rise of both the anticipatory ($\rho_{(55)} = 0.78$, $p < 0.001$) and reactive ($\rho_{(57)} = 0.35$, $p = 0.0078$) process were positively associated with total earnings. In addition, the baseline probability of the anticipatory process to be active ($\rho_{(50)} = 0.72$, $p < 0.001$), as well as the speed at which this probability rose over successive trials ($\rho_{(49)} = 0.52$, $p < 0.001$) were both positively correlated to total earnings. None of the other five model parameters (σ_A, σ_R, effect of a_{n-1} on t_0, effect of R_0 on μ_A, effect of λ on μ_A) showed any significant relationship to total earnings. Thus, two features of the model were particularly rewarded: (1) maximising the speed of evidence accumulation in favour of a manual response (for both the anticipatory and reactive process), and (2) maximising the probability of the anticipatory process to be active.

Finally, we used the model developed above to investigate the mechanism through which impulsivity led to a functional advantage on the TLT. This was done by computing the Spearman correlation between Impulsivity Score and the four functional model parameter estimates (outliers removed as above), while controlling for Age, Gender, and Apathy Score. The only significant correlation was observed between Impulsivity Score and the baseline probability of the anticipatory process to be active ($\rho_{(50)} = 0.24$, $p = 0.045$, one-tail). Note that this is a less sensitive analysis compared to the LMM reported in the main text due to the non-normal distribution of model parameter estimates that obliged us to exclude a large number of data points ($n = 8$ participants excluded from this analysis). Nevertheless, the results were consistent in that they indicated that the functional advantage impulsive individuals benefited from was related to a pre-existing tendency to produce an early anticipatory response before any exposure to the task, rather than any form of learning or sensitivity to the task environment.

Further support for this interpretation was provided by the relationship to questionnaire sub-scales. The only impulsivity sub-scale that showed a significant relationship to the baseline w was the UPPS-P "Lack of Premeditation" ($\rho_{(50)} = 0.30$, $p = 0.019$, one-tail).

Apathy is expressed as a decoupling of reward and fatigue

Lower motivation has been proposed to be a central feature of both apathy and fatigue[6–8]. More specifically, both constructs have been associated with distorted effort-based decision-making[9], and appear to implicate similar neural circuits involved in valuation and reward processing[10–12]. In the present study, fatigue ratings were collected in order to track the development of fatigue throughout the experiment. Participants were asked to rate, on a visual analogue scale (VAS), how tired they felt four times per TLT block (i.e., every 13 trials, see *Methods*).

There was a clear monotonic increase in block-averaged ratings as a function of task progression (Main effect of block on block-averaged fatigue rating: $\beta = 0.28$, $95\%CI = [0.20, 0.38]$, $t_{(478)} = 6.17$, $p < 0.001$; Fig. S9a). Concurrently, and as reported in the main text, the average vigour of the response gradually diminished (Main effect of log-transformed block on block-averaged vigour: $\beta = -0.19$, $95\%CI = [-0.25, -0.12]$, $t_{(478)} = -5.61$, $p < 0.001$), while participants learnt to harvest more reward per trial (Main effect of log-transformed block on block-averaged earnings: $\beta = 0.39$, $95\%CI = [0.32, 0.46]$, $t_{(478)} = 10.72$, $p < 0.001$; Fig. S9a).

A LMM was used to investigate the extent to which block-averaged fatigue ratings co-varied with time-on-task, force exerted (block-averaged vigour), and reward obtained (block-averaged), while controlling for baseline (pre-TLT) fatigue (LMM S9). Higher fatigue was associated with longer time-on-task (Main effect of time-on-task on fatigue ratings: $\beta = 0.34$, $95\%CI = [0.23, 0.45]$, $t_{(475)} = 6.02$, $p < 0.001$; Table S6; Fig. S9) and greater force exertion (Main effect of block vigour on fatigue ratings: $\beta = 0.08$, $95\%CI = [0.01, 0.15]$, $t_{(475)} = 2.33$, $p = 0.020$; Table S6; Fig. S9). By contrast, fatigue showed a negative association with reward (Main effect of block earnings on fatigue ratings: $\beta = -0.07$, $95\%CI = [-0.12, -0.02]$, $t_{(475)} = -2.68$, $p = 0.0076$; Table S6). This finding is consistent with a protective effect of reward on fatigue – as observed in dedicated tasks[13,14] and naturalistic work environments[15,16]. However, the correlational nature of the data did not allow us to conclude on the directionality of the relationship observed. An alternative interpretation is that lower momentary fatigue might have led to better performance and therefore higher reward. Future work should disentangle between these two alternative interpretations.

Finally, adding the two questionnaire-derived scores to the LMM (while controlling for Age and Gender; LMM S10) suggested that apathy weakened the association between earning and fatigue (Interaction Apathy × Block reward: $\beta = 0.06$, $95\%CI = [0.00, 0.14]$, $t_{(466)} = 1.92$, $p = 0.055$; Table S6 – Model 2). That is, in more motivated individuals reward appeared to be negatively associated with fatigue, while in more apathetic participants this effect was much weaker (Fig. S9c). Although this result was not statistically significant, it was considered of interest because it was consistent with apathy promoting of form of fatigue accumulation through a lack of reward sensitivity, a mechanism that is in line with current neurocomputational models[7,8].

Follow-up parcellation of this result into the individual sub-scales of the AMI questionnaire revealed that it was driven mostly by behavioural apathy (Interaction AMI-behavioural × Block reward: $\beta = 0.07$, $95\%CI = [0.00, 0.13]$, $t_{(466)} = 2.03$, $p = 0.043$; Table S7). Fig. S9c illustrates our proposed interpretation of the findings. Apathy is thought to be manifested as a weakening of the protective effect of reward on fatigue accumulation.

References

1. Bueti, D., Bahrami, B. & Walsh, V. Sensory and association cortex in time perception. *Journal of Cognitive Neuroscience* **20**, 1054–1062 (2008). URL https://doi.org/10.1162/jocn.2008.20060.
2. Burnett Heyes, S. *et al.* Impulsivity and rapid decision-making for reward. *Frontiers in Psychology* **3**, 153 (2012). URL https://www.frontiersin.org/article/10.3389/fpsyg.2012.00153.
3. Adam, R., Bays, P. M. & Husain, M. Rapid decision-making under risk. *Cognitive Neuroscience* **3**, 52–61 (2012). URL https://doi.org/10.1080/17588928.2011.613988.
4. Adam, R. *et al.* Dopamine reverses reward insensitivity in apathy following globus pallidus lesions. *Cortex* **49**, 1292 – 1303 (2013). URL http://www.sciencedirect.com/science/article/pii/S0010945212001402.
5. Reddi, B. A. J. & Carpenter, R. H. S. The influence of urgency on decision time. *Nature Neuroscience* **3**, 827–830 (2000). URL https://doi.org/10.1038/77739.

6. Dobryakova, E., DeLuca, J., Genova, H. M. & Wylie, G. R. Neural correlates of cognitive fatigue: Cortico-striatal circuitry and effort–reward imbalance. *Journal of the International Neuropsychological Society* **19**, 849–853 (2013). URL https://doi.org/10.1017/S1355617713000684.
7. Müller, T. & Apps, M. A. Motivational fatigue: A neurocognitive framework for the impact of effortful exertion on subsequent motivation. *Neuropsychologia* **123**, 141–151 (2019). URL https://www.sciencedirect.com/science/article/pii/S0028393218301714. Cognitive Effort.
8. Husain, M. & Roiser, J. P. Neuroscience of apathy and anhedonia: A transdiagnostic approach. *Nature Reviews Neuroscience* **19**, 470–484 (2018). URL http://dx.doi.org/10.1038/s41583-018-0029-9.
9. Jurgelis, M. *et al.* Heightened effort discounting is a common feature of both apathy and fatigue. *Scientific Reports* **11**, 22283 (2021). URL https://doi.org/10.1038/s41598-021-01287-2.
10. Soutschek, A. & Tobler, P. N. Causal role of lateral prefrontal cortex in mental effort and fatigue. *Human Brain Mapping* **41**, 4630–4640 (2020). URL https://onlinelibrary.wiley.com/doi/abs/10.1002/hbm.25146.
11. Hogan, P. S., Chen, S. X., Teh, W. W. & Chib, V. S. Neural mechanisms underlying the effects of physical fatigue on effort-based choice. *Nature Communications* **11**, 4026 (2020). URL https://doi.org/10.1038/s41467-020-17855-5.
12. Müller, T., Klein-Flügge, M. C., Manohar, S. G., Husain, M. & Apps, M. A. J. Neural and computational mechanisms of momentary fatigue and persistence in effort-based choice. *Nature Communications* **12**, 4593 (2021). URL https://doi.org/10.1038/s41467-021-24927-7.
13. Dobryakova, E. *et al.* Fronto-striatal network activation leads to less fatigue in multiple sclerosis. *Multiple Sclerosis Journal* **24**, 1174–1182 (2018). URL https://doi.org/10.1177/1352458517717087.
14. Dobryakova, E. *et al.* Reward presentation reduces on-task fatigue in traumatic brain injury. *Cortex* **126**, 16–25 (2020). URL https://www.sciencedirect.com/science/article/pii/S0010945220300228.
15. Beckers, D. G. *et al.* Voluntary or involuntary? control over overtime and rewards for overtime in relation to fatigue and work satisfaction. *Work & Stress* **22**, 33–50 (2008). URL https://doi.org/10.1080/02678370801984927.
16. Johnston, D. W. *et al.* Why does work cause fatigue? A real-time investigation of fatigue, and determinants of fatigue in nurses working 12-hour shifts. *Annals of Behavioral Medicine* **53**, 551–562 (2018). URL https://doi.org/10.1093/abm/kay065.

Supplementary figures

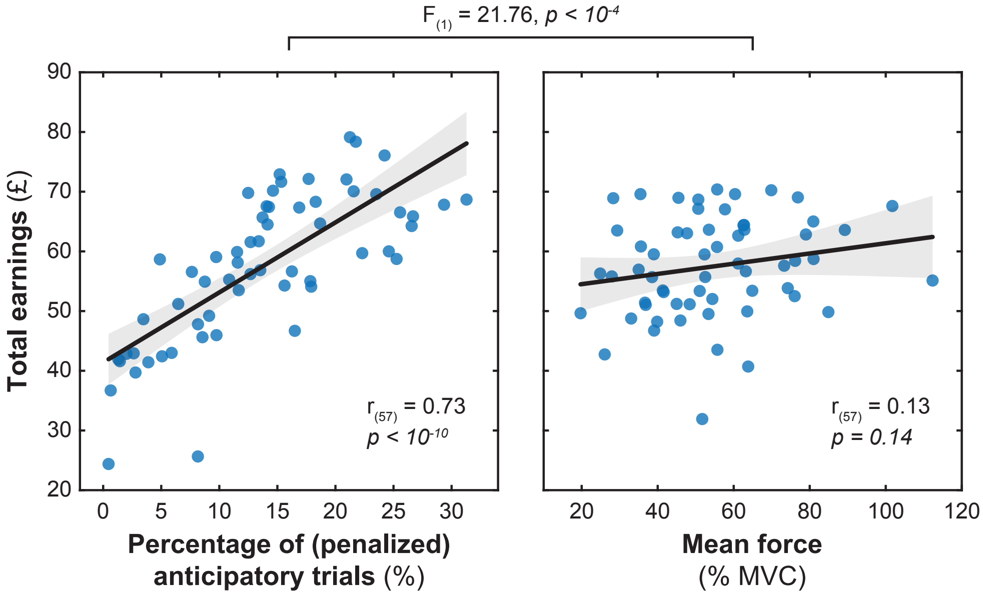

Figure S1. Anticipatory but not strong responses are functionally advantageous. A multiple linear regression showed that total earnings were best predicted by the number of (penalised) anticipatory trials, than the average force exerted during the response (Tables S1 – Model 1).

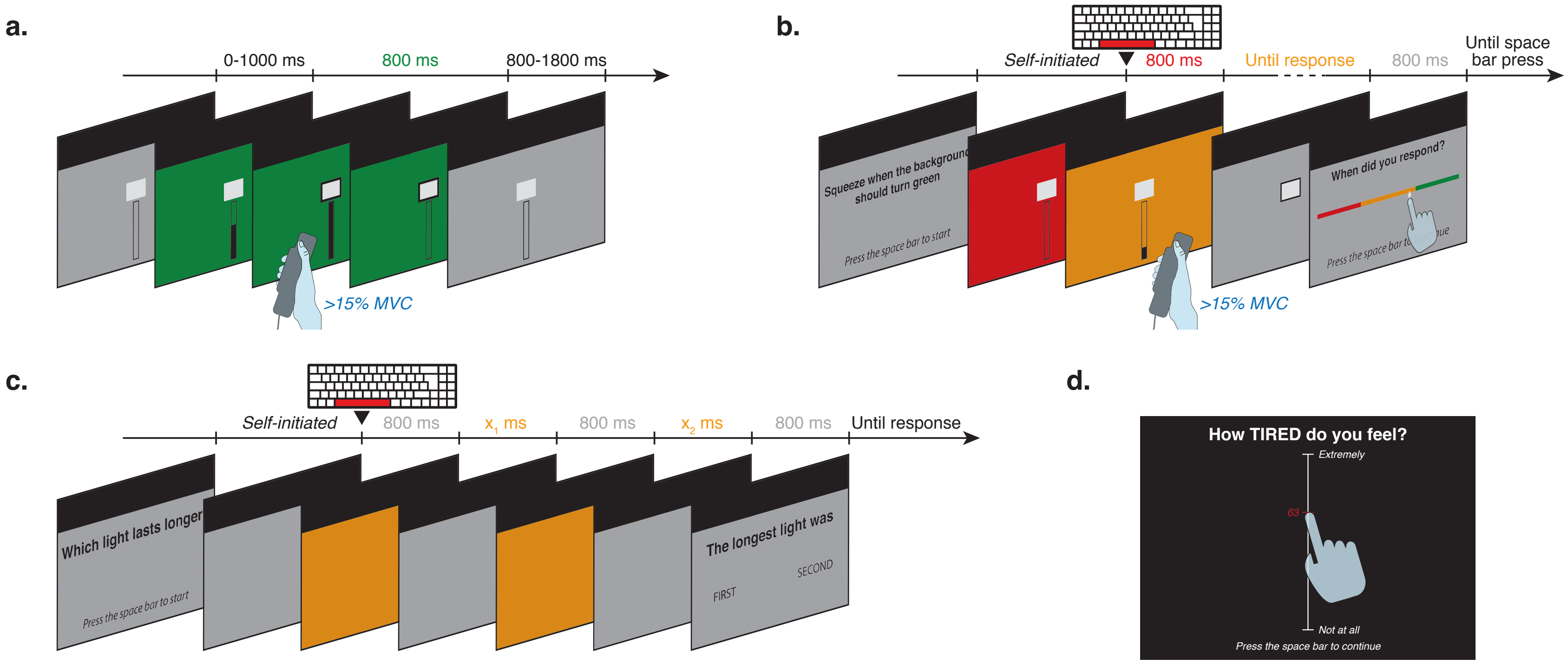

Figure S2. Control tasks. a. On the Reactive Control Task, participants were instructed to respond to unpredictable changes in background colour from grey to green. This task was performed before the Traffic Light Task (TLT), and also served as a training to the use of the dynamometer. **b.** On the Predictive Control Task, the background turned red then amber, and participants were instructed the respond when they thought the background should turn green (based on their experience of the TLT). After their response, a visual representation of the normal time line was displayed, and patient had to indicate (by touching the screen to position an arrow) when they thought they had responded in relation to this time line. This control task was interleaved with the TLT (10 trials at the enf of each TLT block). **c.** On the Temporal Duration Discrimination Task, the back ground turned amber twice in a row, and participants had to indicate which of these two flashes (the first or the second) lasted the longest. This control task was performed last, after the TLT. **d.** At various points throughout the experimental session, participants were asked to rate how tired they felt. When doing so, a vertical scale appeared in the middle of the screen, and participants could place a cursor at the appropriate level. A number between 0 (no fatigue at all) and 100 (extremely tired) was displayed on the side of the cursor based on its position on the scale.

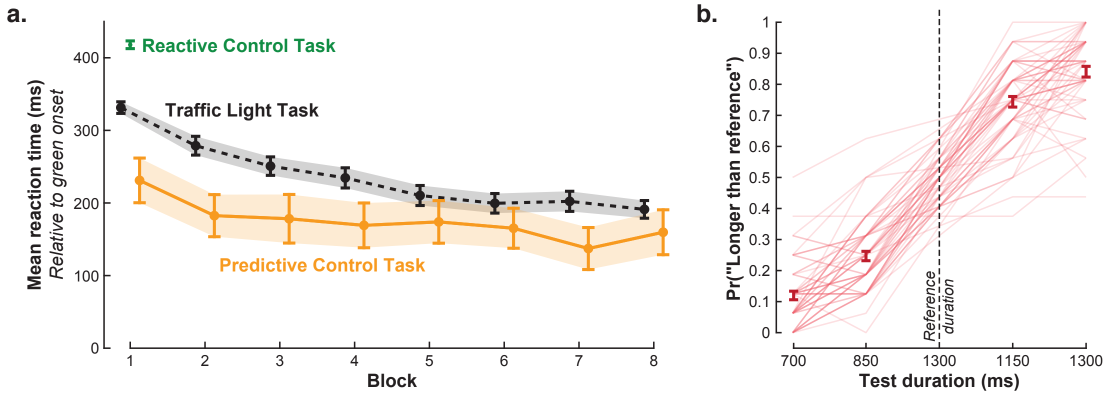

Figure S3. Performance on control tasks. a. Mean reaction time as a function of experimental block on the Traffic Light Task (dotted black line) and the Predictive Control Task (full orange line). Mean reaction time on the Reactive Control task is plotted for reference in green. Error bars represent s.e.m. **b.** Probability of responding that the test duration lasted longer than the reference duration as a function of test duration. Error bars represent s.e.m. Lines represent individual participants.

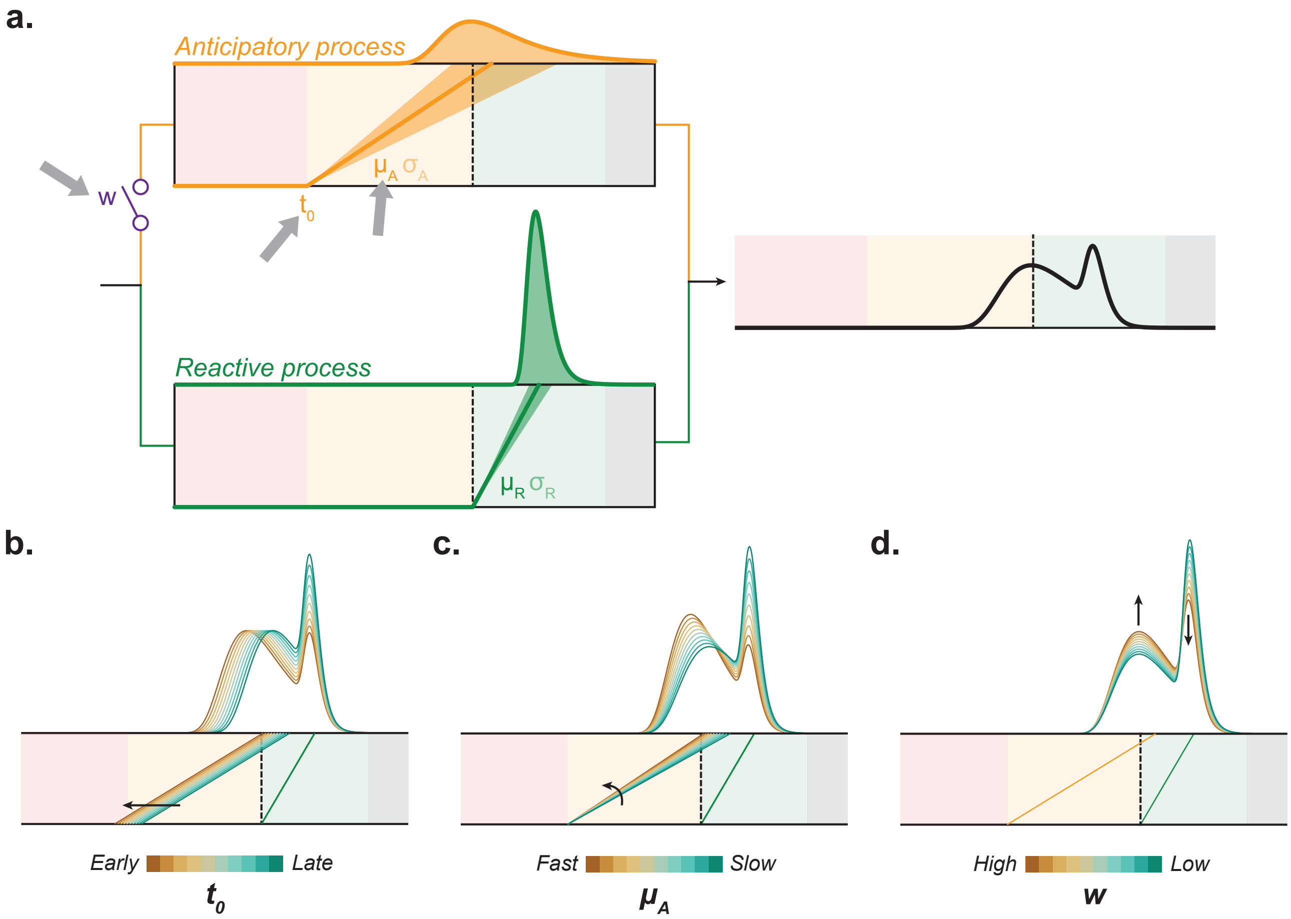

Figure S4. Visual illustration of the two-horse linear rise-to-threshold model of reaction time in TLT. a. Group and individual reaction time data were fit to a two-horse linear rise-to-threshold model. RT distributions arise probabilistically from one of two linear rise-to-threshold processes: (1) an anticipatory decision process, triggered by the onset of the amber light, with mean rate of rise mu_A and SD rate of rise $sigma_A$. The background in the box shows the duration of red, amber and green lights. The dashed vertical line marks the onset of the green light. This process has a small mean rate of rise and large variance, and therefore results in a wide but early RT distribution (the orange distribution above the top box), of which some are elicited prior to the onset of the green light. (2) A reactive decision process, triggered by the onset of the green light, with mean rate of rise mu_R and SD rate of rise $sigma_R$. This process has a high mean rate of rise and small variance, and therefore results in a narrow but late RT distribution with median shortly after green onset (the green distribution above the bottom box). The first process to reach the threshold (the top horizontal edge of each box) on a given trial will trigger a response. This results in a two-peak RT distribution observed in TLT, as illustrated in blue on the right box. The anticipatory process can be affected in three mechanisms: **b.** the starting point of the anticipatory process t_0; **c.** the mean rise of the anticipatory process μ_A, and **d.** the probability of activating the anticipatory process w.

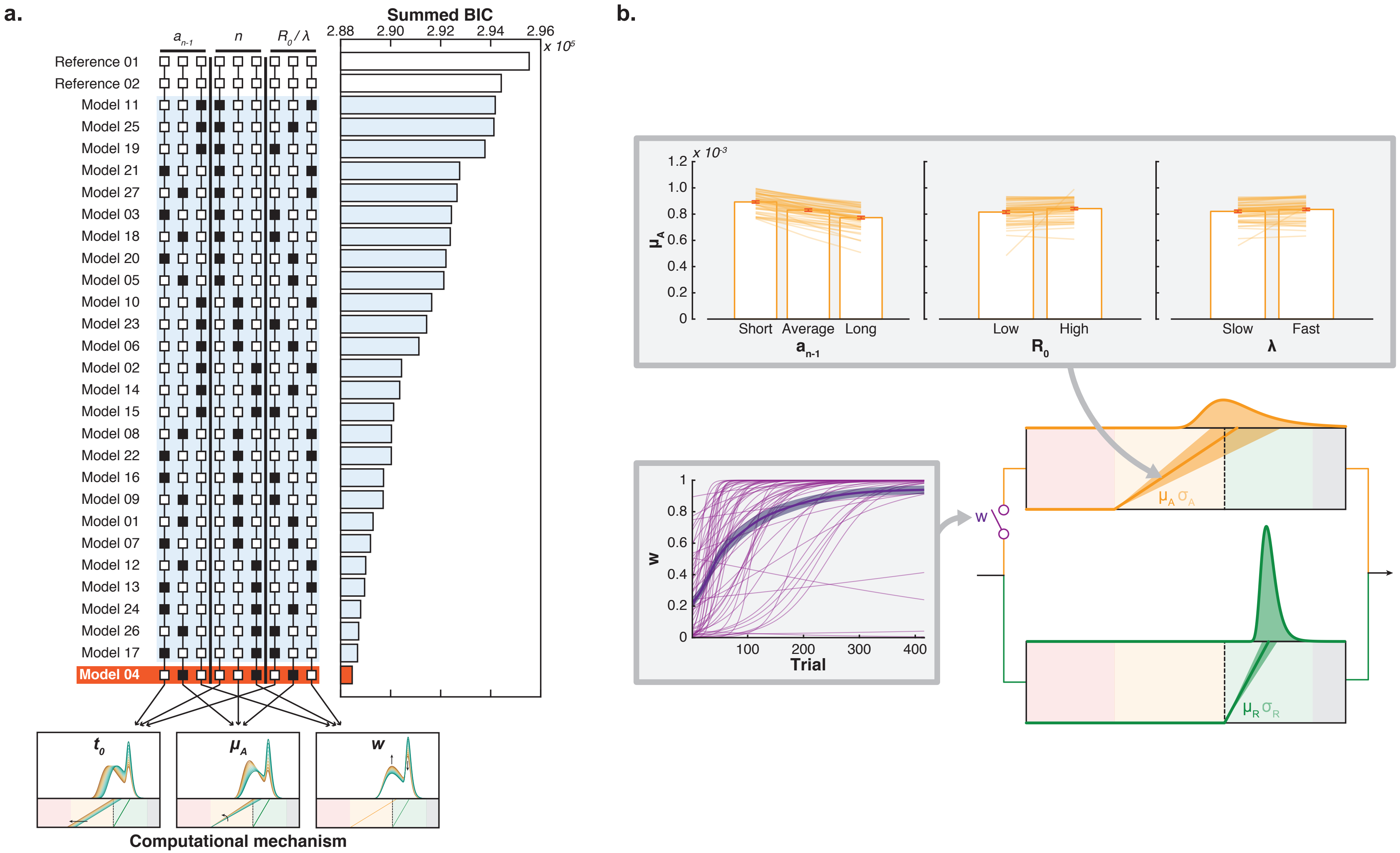

Figure S5. Model comparison and winning model. a. The Bayesian Information Criterion (BIC) summed across all participants is plotted for every model tested. The box grid shows the model architecture. Reference models 1 and 2 were a two-horse and weighted two-horse model, respectively. Models 1-27 were constructed by varying the computational mechanisms (effect on t_0, μ_A or w) by which each experimental factor (a_{n-1}, n, $R0/\lambda$) had an effect. For each of the three columns, a black box on the left/middle/right means an effect on $t_0/\mu_A/w$, respectively. The best model (lowest BIC) included an effect of a_{n-1} and cost-benefit structure (R_0, λ) on μ_A, and an effect of trial number on w. **b.** These effects are plotted here. Bars and error bars indicate group mean ± s.e.m. (outliers removed). Individual participants are shown as individual lines. a_{n-1}: duration of the amber light in the previous trial; n: trial index; R_0: intercept of the reward function; λ: time constant of the reward function; μ_A: mean rate-of-rise of the anticipatory process; w: probability of the anticipatory process to be active.

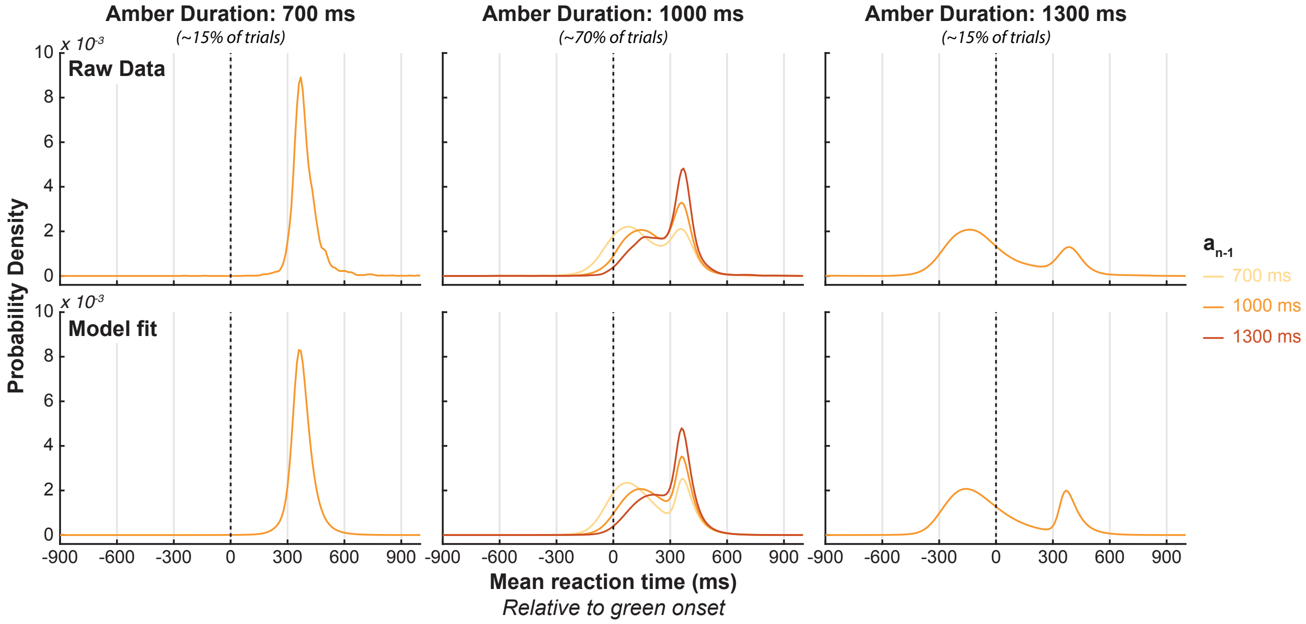

Figure S6. Observed and modeled reaction time distributions, partitioned by amber duration of the current and previous trial. From left to right, each column shows the RT distribution (relative to green onset) pooled across participants for the short (700 ms), average (1000 ms) and long (1300 ms) amber duration. The top row shows the raw data (smoothed with a normal kernel function and estimated every 10 ms), and the bottom one shows the fit of the winning model (model 4). The line colour represents the duration of the amber light in the previous trial (a_{n-1}). Note that, by design, an extreme amber duration was always followed by an average one. This explains why the a_{n-1} partitioning is only effective for a current amber duration of 1000 ms. The best account of the influence of a_{n-1} on the RT distribution implicated an effect on the mean rate-of-rise of the anticipatory process (μ_A; see Fig. S5).

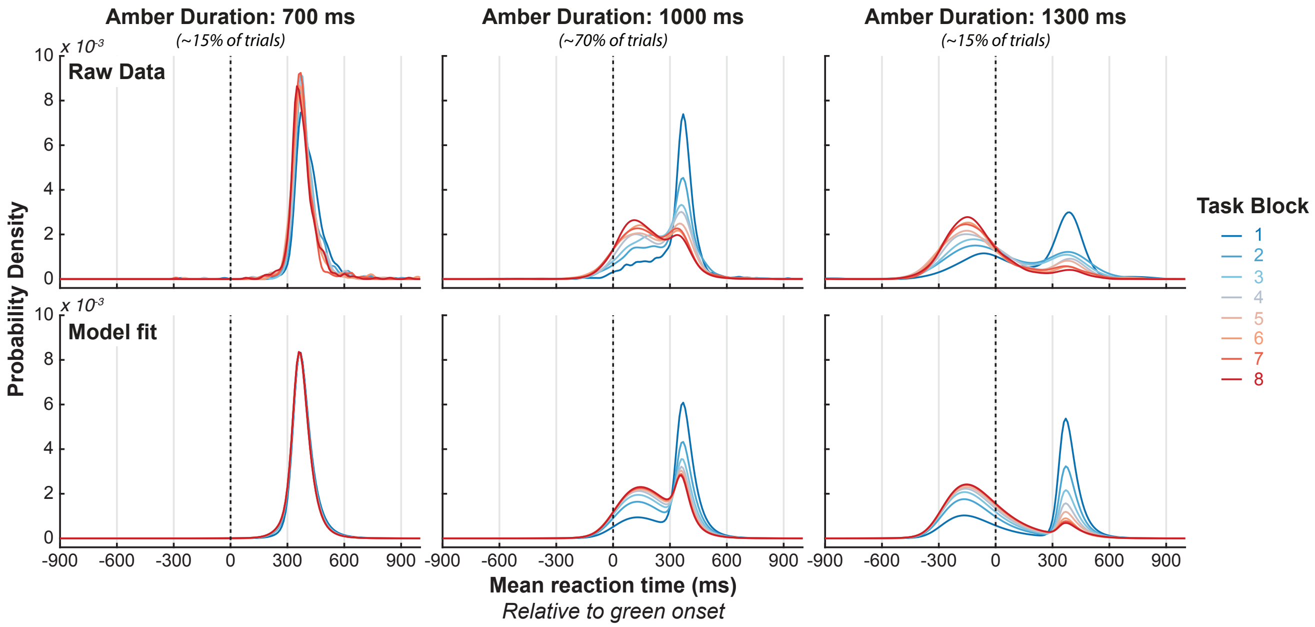

Figure S7. Observed and modeled reaction time distributions, partitioned by amber duration and block index. From left to right, each column shows the RT distribution (relative to green onset) pooled across participants for the short (700 ms), average (1000 ms) and long (1300 ms) amber duration. The top row shows the raw data (smoothed with a normal kernel function and estimated every 10 ms), and the bottom one shows the fit of the winning model (model 4). The line colour represents the task block number. The best account of this learning effect implicated an effect of trial index on the probability of the anticipatory process to be active (w; see Fig. S5).

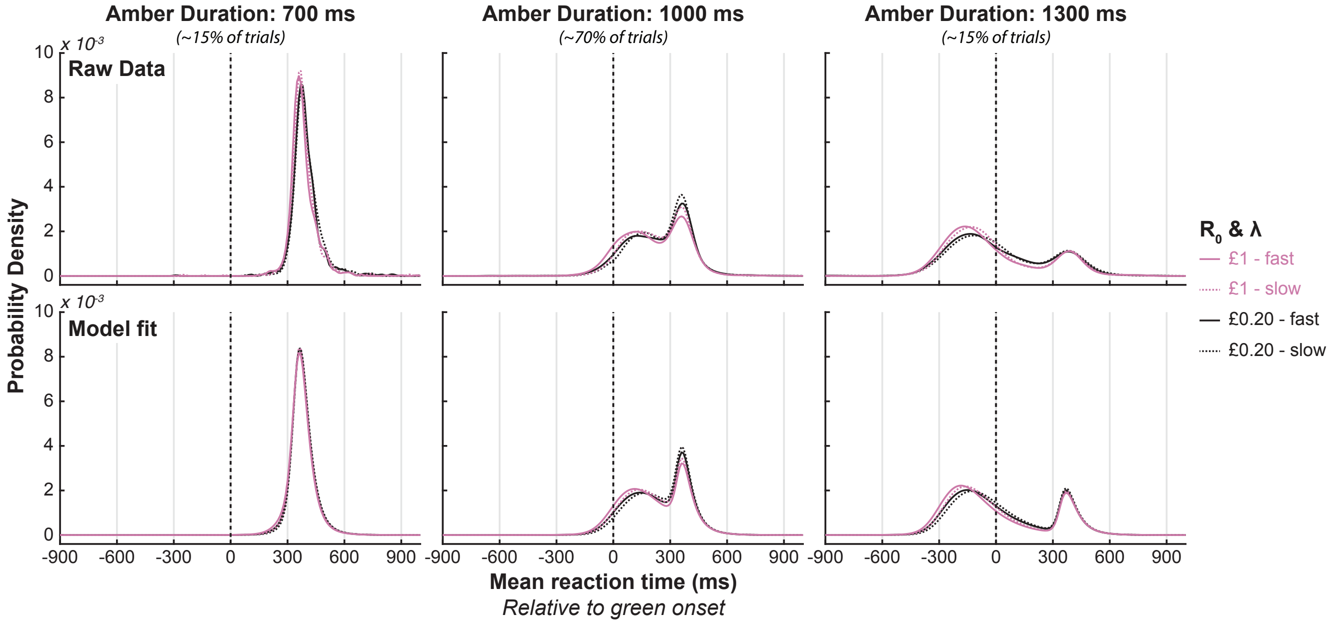

Figure S8. Observed and modeled reaction time distributions, partitioned by amber duration and cost-benefit information. From left to right, each column shows the RT distribution (relative to green onset) pooled across participants for the short (700 ms), average (1000 ms) and long (1300 ms) amber duration. The top row shows the raw data (smoothed with a normal kernel function and estimated every 10 ms), and the bottom one shows the fit of the winning model (model 4). The line colour represents the amount of reward available (pink: $R_0 = £1$; black: $R_0 = £0.20$). Solid lines represent trials in which the reward decayed quickly after green onset ($\lambda = 0.0125$ ms^{-1}), and dotted lines represent trials in which it decayed more slowly ($\lambda = 0.0033$ ms^{-1}). The best account of the effect of the cost-benefit structure implicated an effect of R_0 and λ on the mean rate-of-rise of the anticipatory process (μ_A; see Fig. S5).

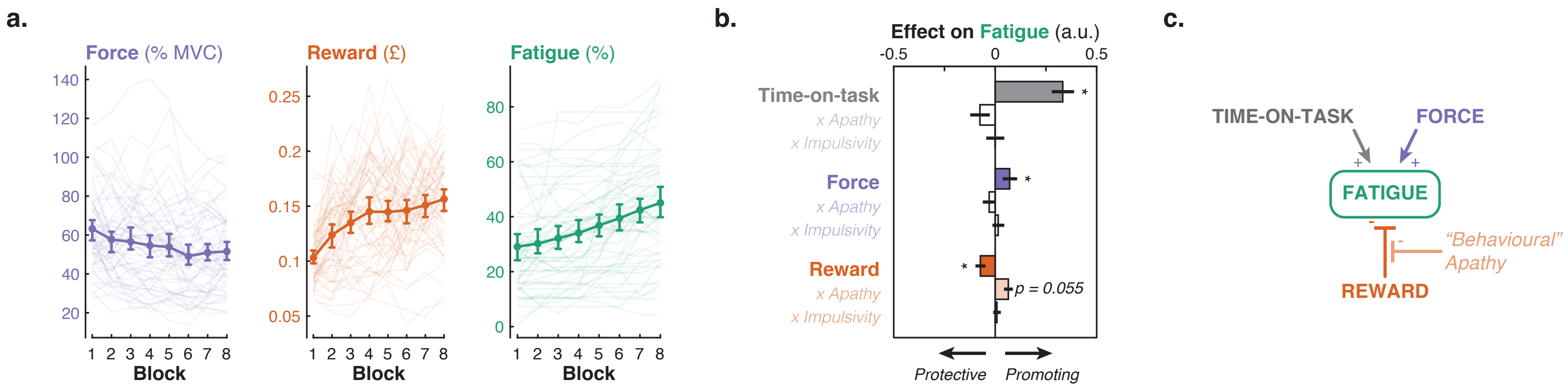

Figure S9. Apathy reduces the protective effect of reward on fatigue accumulation. a. As participants progressed through the task, the vigour of their response decreased (left graph) while the average reward obtained per trial increased (middle graph). Simultaneously, fatigue ratings showed a marked monotonic increase (right graph). For each TLT block, Points and error bars show group mean with boot-strapped 95% confidence intervals. Individual participants are shown as thin lines. **b.** Parameter estimates of the linear mixed-effect model of fatigue (see also Table S6 – Model 2). Coloured bars show significant effects ($p < 0.05$) and white bars non significant effects with standard error. Longer time-on-task and higher force exertion were associated with higher fatigue ratings. By contrast, higher average reward was linked to lower fatigue. The latter was counteracted by apathy. Asterisks indicate significance ($p < 0.05$). **c.** Graphical representation of the proposed interpretation of the finding.

Supplementary tables

	**Model 1**	**Model 2**
(Intercept)	$\beta = -4.36 \times 10^{-16}$<br>$SE = 0.0831$<br>$t_{57} = -0.00$<br>$p = 1.00$	$\beta = -0.0281$<br>$SE = 0.223$<br>$t_{56} = -0.13$<br>$p = 0.90$
Anticipation	$\beta = +0.734$<br>$SE = 0.0844$<br>$t_{57} = +8.70$<br>***p<0.0001***	
Vigour	$\beta = +0.131$<br>$SE = 0.0844$<br>$t_{57} = +1.55$<br>$p = 0.13$	
Age		$\beta = +0.0925$<br>$SE = 0.12$<br>$t_{56} = +0.77$<br>$p = 0.45$
Apathy Score		$\beta = -0.0766$<br>$SE = 0.143$<br>$t_{56} = -0.54$<br>$p = 0.59$
Impulsivity Score		$\beta = +0.218$<br>$SE = 0.0962$<br>$t_{56} = +2.26$<br>***p = 0.027***
N_{obs}	60	60
$adj-R^2$	0.56	0.12
BIC	158.40	222.12

Table S1. Fixed effects of the analysis of total earnings. Models were specified as follows. Model 1: Earnings $\sim$ 1 + Anticipation + Vigour + (1 + Anticipation + Vigour | Gender); Model 2: Earnings $\sim$ 1 + Apathy Score + Impulsivity Score + Age + (1 + Apathy Score + Impulsivity Score + Age | Gender).

	Model 1	Model 2
(Intercept)	$\beta = +0.000134$<br>$SE = 0.0591$<br>$t_{9294} = +0.00$<br>$p = 1.00$	$\beta = +0.0149$<br>$SE = 0.108$<br>$t_{9282} = +0.14$<br>$p = 0.89$
R_0	$\beta = -0.0634$<br>$SE = 0.0104$<br>$t_{9294} = -6.12$<br>***$p<0.0001$***	$\beta = -0.0635$<br>$SE = 0.0138$<br>$t_{9282} = -4.59$<br>***$p<0.0001$***
λ	$\beta = -0.0359$<br>$SE = 0.00941$<br>$t_{9294} = -3.82$<br>***$p = 0.00014$***	$\beta = -0.0348$<br>$SE = 0.00914$<br>$t_{9282} = -3.81$<br>***$p = 0.00014$***
a_n	$\beta = -0.621$<br>$SE = 0.0275$<br>$t_{9294} = -22.59$<br>***$p<0.0001$***	$\beta = -0.613$<br>$SE = 0.0414$<br>$t_{9282} = -14.82$<br>***$p<0.0001$***
a_{n-1}	$\beta = +0.177$<br>$SE = 0.00905$<br>$t_{9294} = +19.51$<br>***$p<0.0001$***	$\beta = +0.175$<br>$SE = 0.00847$<br>$t_{9282} = +20.72$<br>***$p<0.0001$***
log(Block)	$\beta = -0.23$<br>$SE = 0.0191$<br>$t_{9294} = -12.06$<br>***$p<0.0001$***	$\beta = -0.226$<br>$SE = 0.0285$<br>$t_{9282} = -7.93$<br>***$p<0.0001$***
λ:R_0	$\beta = -0.00969$<br>$SE = 0.00578$<br>$t_{9294} = -1.68$<br>$p = 0.09$	
Age		$\beta = -0.0133$<br>$SE = 0.0471$<br>$t_{9282} = -0.28$<br>$p = 0.78$
a_n:Apathy Score		$\beta = +0.0116$<br>$SE = 0.0352$<br>$t_{9282} = +0.33$<br>$p = 0.74$
a_n:Impulsivity Score		$\beta = -0.0502$<br>$SE = 0.0204$<br>$t_{9282} = -2.46$<br>***$p = 0.014$***
a_{n-1}:Apathy Score		$\beta = -0.0175$<br>$SE = 0.00989$<br>$t_{9282} = -1.77$<br>$p = 0.08$
a_{n-1}:Impulsivity Score		$\beta = +0.0147$<br>$SE = 0.0089$<br>$t_{9282} = +1.65$<br>$p = 0.10$
Apathy Score		$\beta = +0.0308$<br>$SE = 0.0921$<br>$t_{9282} = +0.33$<br>$p = 0.74$
Apathy Score:R_0		$\beta = +0.0106$<br>$SE = 0.0088$<br>$t_{9282} = +1.20$<br>$p = 0.23$
Apathy Score:λ		$\beta = -0.0147$<br>$SE = 0.00888$<br>$t_{9282} = -1.65$<br>$p = 0.10$
Apathy Score:log(Block)		$\beta = +0.00739$<br>$SE = 0.0239$<br>$t_{9282} = +0.31$<br>$p = 0.76$
Impulsivity Score		$\beta = -0.0987$<br>$SE = 0.0433$<br>$t_{9282} = -2.28$<br>***$p = 0.023$***
Impulsivity Score:R_0		$\beta = -0.00285$<br>$SE = 0.00888$<br>$t_{9282} = -0.32$<br>$p = 0.75$
Impulsivity Score:λ		$\beta = +0.00429$<br>$SE = 0.00737$<br>$t_{9282} = +0.58$<br>$p = 0.56$
Impulsivity Score:log(Block)		$\beta = -0.00238$<br>$SE = 0.0195$<br>$t_{9282} = -0.12$<br>$p = 0.90$
N_{obs}	9301	9301
$adj-R^2$	0.76	0.76
BIC	14252.13	15999.69

Table S2. Fixed effects of the analysis of mean reaction time. Models were specified as follows. Model 1: Mean RT $\sim$ 1 + a_{n-1} + a_n + log(Block) + λ*R_0 + (1 + a_{n-1} + a_n + log(Block) + λ*R_0 | participant); Model 2: Mean RT $\sim$ 1 + Age + a_{n-1}*Apathy Score + a_n*Apathy Score + a_{n-1}*Impulsivity Score + a_n*Impulsivity Score + Apathy Score*λ + Impulsivity Score*λ + Apathy Score*R_0 + Impulsivity Score*R_0 + Apathy Score*log(Block) + Impulsivity Score*log(Block) + (1 + Age + a_{n-1}*Apathy Score + a_n*Apathy Score + a_{n-1}*Impulsivity Score + a_n*Impulsivity Score + Apathy Score*λ + Impulsivity Score*λ + Apathy Score*R_0 + Impulsivity Score*R_0 + Apathy Score*log(Block) + Impulsivity Score*log(Block) | Gender) + (1 + a_{n-1} + a_n + λ + R_0 + log(Block) | participant).

	Model 1	Model 2
(Intercept)	$\beta = -0.00606$<br>$SE = 0.0942$<br>$t_{9281} = -0.06$<br>$p = 0.95$	$\beta = -0.00495$<br>$SE = 0.108$<br>$t_{9285} = -0.05$<br>$p = 0.96$
Age	$\beta = -0.0371$<br>$SE = 0.0551$<br>$t_{9281} = -0.67$<br>$p = 0.50$	$\beta = -0.0281$<br>$SE = 0.054$<br>$t_{9285} = -0.52$<br>$p = 0.60$
R_0	$\beta = -0.0633$<br>$SE = 0.0131$<br>$t_{9281} = -4.84$<br>***p<0.0001***	$\beta = -0.0633$<br>$SE = 0.0131$<br>$t_{9285} = -4.84$<br>***p<0.0001***
λ	$\beta = -0.036$<br>$SE = 0.00948$<br>$t_{9281} = -3.79$<br>***p = 0.00015***	$\beta = -0.0359$<br>$SE = 0.00948$<br>$t_{9285} = -3.79$<br>***p = 0.00015***
λ:R_0	$\beta = -0.00966$<br>$SE = 0.0123$<br>$t_{9281} = -0.79$<br>$p = 0.43$	$\beta = -0.00974$<br>$SE = 0.0123$<br>$t_{9285} = -0.79$<br>$p = 0.43$
a_n	$\beta = -0.618$<br>$SE = 0.0361$<br>$t_{9281} = -17.10$<br>***p<0.0001***	$\beta = -0.617$<br>$SE = 0.0417$<br>$t_{9285} = -14.80$<br>***p<0.0001***
a_n:Impulsivity Score	$\beta = -0.0756$<br>$SE = 0.0442$<br>$t_{9281} = -1.71$<br>$p = 0.09$	$\beta = +0.0348$<br>$SE = 0.0467$<br>$t_{9285} = +0.74$<br>$p = 0.46$
a_{n-1}	$\beta = +0.177$<br>$SE = 0.00925$<br>$t_{9281} = +19.10$<br>***p<0.0001***	$\beta = +0.177$<br>$SE = 0.00923$<br>$t_{9285} = +19.13$<br>***p<0.0001***
log(Block)	$\beta = -0.23$<br>$SE = 0.0282$<br>$t_{9281} = -8.15$<br>***p<0.0001***	$\beta = -0.23$<br>$SE = 0.0282$<br>$t_{9285} = -8.16$<br>***p<0.0001***
Impulsivity Score	$\beta = -0.0964$<br>$SE = 0.0884$<br>$t_{9281} = -1.09$<br>$p = 0.28$	$\beta = +0.00336$<br>$SE = 0.115$<br>$t_{9285} = +0.03$<br>$p = 0.98$
UPPS-P$_{\text{Persev}}$	$\beta = -0.0179$<br>$SE = 0.065$<br>$t_{9281} = -0.27$<br>$p = 0.78$	
UPPS-P$_{\text{Premed}}$	$\beta = +0.0821$<br>$SE = 0.0498$<br>$t_{9281} = +1.65$<br>$p = 0.10$	
UPPS-P$_{\text{negU}}$	$\beta = +0.114$<br>$SE = 0.063$<br>$t_{9281} = +1.80$<br>$p = 0.07$	
UPPS-P$_{\text{posU}}$	$\beta = -0.128$<br>$SE = 0.0622$<br>$t_{9281} = -2.06$<br>***p = 0.039***	
UPPS-P$_{\text{SS}}$	$\beta = +0.079$<br>$SE = 0.0392$<br>$t_{9281} = +2.01$<br>***p = 0.044***	
a_n:UPPS-P$_{\text{Persev}}$	$\beta = +0.0239$<br>$SE = 0.0294$<br>$t_{9281} = +0.81$<br>$p = 0.42$	
a_n:UPPS-P$_{\text{Premed}}$	$\beta = +0.0235$<br>$SE = 0.0228$<br>$t_{9281} = +1.03$<br>$p = 0.30$	
a_n:UPPS-P$_{\text{negU}}$	$\beta = +0.0752$<br>$SE = 0.0271$<br>$t_{9281} = +2.78$<br>***p = 0.0055***	
a_n:UPPS-P$_{\text{posU}}$	$\beta = -0.0638$<br>$SE = 0.0312$<br>$t_{9281} = -2.05$<br>***p = 0.041***	
a_n:UPPS-P$_{\text{SS}}$	$\beta = +0.0484$<br>$SE = 0.018$<br>$t_{9281} = +2.68$<br>***p = 0.0073***	
BIS$_{\text{att}}$		$\beta = -0.00134$<br>$SE = 0.0566$<br>$t_{9285} = -0.02$<br>$p = 0.98$
BIS$_{\text{mot}}$		$\beta = -0.0471$<br>$SE = 0.0602$<br>$t_{9285} = -0.78$<br>$p = 0.43$
BIS$_{\text{np}}$		$\beta = -0.024$<br>$SE = 0.093$<br>$t_{9285} = -0.26$<br>$p = 0.80$
a_n:BIS$_{\text{att}}$		$\beta = +0.00692$<br>$SE = 0.03$<br>$t_{9285} = +0.23$<br>$p = 0.82$
a_n:BIS$_{\text{mot}}$		$\beta = -0.036$<br>$SE = 0.0306$<br>$t_{9285} = -1.18$<br>$p = 0.24$
a_n:BIS$_{\text{np}}$		$\beta = -0.0722$<br>$SE = 0.0363$<br>$t_{9285} = -1.99$<br>***p = 0.047***
N_{obs}	9301	9301
$adj-R^2$	0.76	0.76
BIC	16233.59	15531.06

Table S3. Fixed effects of the analysis of the relationship between questionnaire sub-scales and mean reaction time.
(Legend continued on next page)

Models were specified as follows. Model 1: Mean RT $\sim$ 1 + a_{n-1} + log(Block) + Age + a_n*Impulsivity Score + λ*R_0 + a_n*UPPS-P$_{\text{Persev}}$ + a_n*UPPS-P$_{\text{Premed}}$ + a_n*UPPS-P$_{\text{negU}}$ + a_n*UPPS-P$_{\text{posU}}$ + a_n*UPPS-P$_{\text{SS}}$ + (1 + a_{n-1} + log(Block) + Age + a_n*Impulsivity Score + λ*R_0 + a_n*UPPS-P$_{\text{Persev}}$ + a_n*UPPS-P$_{\text{Premed}}$ + a_n*UPPS-P$_{\text{negU}}$ + a_n*UPPS-P$_{\text{posU}}$ + a_n*UPPS-P$_{\text{SS}}$ | Gender) + (1 + a_{n-1} + a_n + log(Block) + λ*R_0 | participant); Model 2: Mean RT $\sim$ 1 + a_{n-1} + log(Block) + Age + a_n*Impulsivity Score + λ*R_0 + a_n*BIS$_{\text{np}}$ + a_n*BIS$_{\text{mot}}$ + a_n*BIS$_{\text{att}}$ + (1 + a_{n-1} + log(Block) + Age + a_n*Impulsivity Score + λ*R_0 + a_n*BIS$_{\text{np}}$ + a_n*BIS$_{\text{mot}}$ + a_n*BIS$_{\text{att}}$ | Gender) + (1 + a_{n-1} + a_n + log(Block) + λ*R_0 | participant).

	**Model 1**	**Model 2**
(Intercept)	$\beta = +0.0159$<br>$SE = 0.106$<br>$t_{9293} = +0.15$<br>$p = 0.88$	$\beta = +0.0355$<br>$SE = 0.234$<br>$t_{9287} = +0.15$<br>$p = 0.88$
R_0	$\beta = +0.089$<br>$SE = 0.0102$<br>$t_{9293} = +8.68$<br>***p<0.0001***	$\beta = +0.0895$<br>$SE = 0.0211$<br>$t_{9287} = +4.24$<br>***p<0.0001***
a_n	$\beta = -0.0957$<br>$SE = 0.0128$<br>$t_{9293} = -7.48$<br>***p<0.0001***	$\beta = -0.1$<br>$SE = 0.0172$<br>$t_{9287} = -5.83$<br>***p<0.0001***
log(Block)	$\beta = -0.186$<br>$SE = 0.0298$<br>$t_{9293} = -6.24$<br>***p<0.0001***	$\beta = -0.192$<br>$SE = 0.049$<br>$t_{9287} = -3.91$<br>***p<0.0001***
Mean RT	$\beta = -0.104$<br>$SE = 0.0184$<br>$t_{9293} = -5.62$<br>***p<0.0001***	$\beta = -0.11$<br>$SE = 0.0193$<br>$t_{9287} = -5.69$<br>***p<0.0001***
λ	$\beta = -0.00667$<br>$SE = 0.011$<br>$t_{9293} = -0.61$<br>$p = 0.54$	
λ:R_0	$\beta = +0.000975$<br>$SE = 0.00494$<br>$t_{9293} = +0.20$<br>$p = 0.84$	
a_{n-1}	$\beta = -0.000984$<br>$SE = 0.00579$<br>$t_{9293} = -0.17$<br>$p = 0.87$	
Age		$\beta = -0.0692$<br>$SE = 0.108$<br>$t_{9287} = -0.64$<br>$p = 0.52$
a_n:Apathy Score		$\beta = +0.00148$<br>$SE = 0.0108$<br>$t_{9287} = +0.14$<br>$p = 0.89$
a_n:Impulsivity Score		$\beta = -0.0114$<br>$SE = 0.0177$<br>$t_{9287} = -0.65$<br>$p = 0.52$
Apathy Score		$\beta = -0.0837$<br>$SE = 0.0975$<br>$t_{9287} = -0.86$<br>$p = 0.39$
Apathy Score:R_0		$\beta = -0.0175$<br>$SE = 0.00871$<br>$t_{9287} = -2.01$<br>***p = 0.044***
Apathy Score:log(Block)		$\beta = -0.0273$<br>$SE = 0.0322$<br>$t_{9287} = -0.85$<br>$p = 0.40$
Impulsivity Score		$\beta = -0.0745$<br>$SE = 0.0772$<br>$t_{9287} = -0.96$<br>$p = 0.33$
Impulsivity Score:R_0		$\beta = +0.0052$<br>$SE = 0.00745$<br>$t_{9287} = +0.70$<br>$p = 0.49$
Impulsivity Score:log(Block)		$\beta = +0.0176$<br>$SE = 0.0273$<br>$t_{9287} = +0.65$<br>$p = 0.52$
N_{obs}	9301	9301
$adj-R^2$	0.79	0.79
BIC	13233.15	14212.42

Table S4. Fixed effects of the analysis of mean Vigour. Models were specified as follows. Model 1: vigour $\sim$ 1 + a_{n-1} + a_n + Mean RT + log(Block) + λ*R_0 + (1 + a_{n-1} + a_n + Mean RT + log(Block) + λ*R_0 | participant); Model 2: vigour $\sim$ 1 + Mean RT + Age + a_n*Apathy Score + a_n*Impulsivity Score + Apathy Score*R_0 + Impulsivity Score*R_0 + Apathy Score*log(Block) + Impulsivity Score*log(Block) + (1 + Mean RT + Age + a_n*Apathy Score + a_n*Impulsivity Score + Apathy Score*R_0 + Impulsivity Score*R_0 + Apathy Score*log(Block) + Impulsivity Score*log(Block) | Gender) + (1 + a_n + Mean RT + R_0 + log(Block) | participant).

	Model 1	Model 2
(Intercept)	$\beta = +0.0301$<br>$SE = 0.243$<br>$t_{9287} = +0.12$<br>$p = 0.90$	$\beta = +0.099$<br>$SE = 0.223$<br>$t_{9285} = +0.44$<br>$p = 0.66$
Age	$\beta = +0.0432$<br>$SE = 0.128$<br>$t_{9287} = +0.34$<br>$p = 0.73$	$\beta = -0.11$<br>$SE = 0.0953$<br>$t_{9285} = -1.16$<br>$p = 0.25$
R_0	$\beta = +0.09$<br>$SE = 0.0222$<br>$t_{9287} = +4.05$<br>***p<0.0001***	$\beta = +0.0913$<br>$SE = 0.0239$<br>$t_{9285} = +3.82$<br>***p = 0.00013***
a_n	$\beta = -0.0994$<br>$SE = 0.0173$<br>$t_{9287} = -5.75$<br>***p<0.0001***	$\beta = -0.101$<br>$SE = 0.0179$<br>$t_{9285} = -5.64$<br>***p<0.0001***
log(Block)	$\beta = -0.187$<br>$SE = 0.0473$<br>$t_{9287} = -3.96$<br>***p<0.0001***	$\beta = -0.187$<br>$SE = 0.0474$<br>$t_{9285} = -3.95$<br>***p<0.0001***
Mean RT	$\beta = -0.109$<br>$SE = 0.0202$<br>$t_{9287} = -5.41$<br>***p<0.0001***	$\beta = -0.111$<br>$SE = 0.0197$<br>$t_{9285} = -5.64$<br>***p<0.0001***
Impulsivity Score	$\beta = -0.124$<br>$SE = 0.0739$<br>$t_{9287} = -1.67$<br>$p = 0.09$	$\beta = -0.0368$<br>$SE = 0.0788$<br>$t_{9285} = -0.47$<br>$p = 0.64$
Impulsivity Score:R_0	$\beta = -0.00266$<br>$SE = 0.00764$<br>$t_{9287} = -0.35$<br>$p = 0.73$	$\beta = -0.00472$<br>$SE = 0.0108$<br>$t_{9285} = -0.43$<br>$p = 0.66$
AMI_{beh}	$\beta = +0.0538$<br>$SE = 0.182$<br>$t_{9287} = +0.30$<br>$p = 0.77$	
AMI_{emo}	$\beta = -0.247$<br>$SE = 0.0904$<br>$t_{9287} = -2.73$<br>***p = 0.0064***	
AMI_{soc}	$\beta = +0.159$<br>$SE = 0.088$<br>$t_{9287} = +1.81$<br>$p = 0.07$	
R_0:AMI_{beh}	$\beta = -0.00579$<br>$SE = 0.0108$<br>$t_{9287} = -0.54$<br>$p = 0.59$	
R_0:AMI_{emo}	$\beta = -0.0232$<br>$SE = 0.00935$<br>$t_{9287} = -2.48$<br>***p = 0.013***	
R_0:AMI_{soc}	$\beta = -0.00902$<br>$SE = 0.00869$<br>$t_{9287} = -1.04$<br>$p = 0.30$	
AES_{beh}		$\beta = -0.188$<br>$SE = 0.128$<br>$t_{9285} = -1.46$<br>$p = 0.14$
AES_{cog}		$\beta = -0.0627$<br>$SE = 0.208$<br>$t_{9285} = -0.30$<br>$p = 0.76$
AES_{emo}		$\beta = +0.0554$<br>$SE = 0.154$<br>$t_{9285} = +0.36$<br>$p = 0.72$
$\text{AES}_{\text{other}}$		$\beta = -0.0124$<br>$SE = 0.247$<br>$t_{9285} = -0.05$<br>$p = 0.96$
R_0:AES_{beh}		$\beta = +0.00282$<br>$SE = 0.0115$<br>$t_{9285} = +0.25$<br>$p = 0.81$
R_0:AES_{cog}		$\beta = +0.00337$<br>$SE = 0.0114$<br>$t_{9285} = +0.29$<br>$p = 0.77$
R_0:AES_{emo}		$\beta = -0.0302$<br>$SE = 0.021$<br>$t_{9285} = -1.44$<br>$p = 0.15$
R_0:$\text{AES}_{\text{other}}$		$\beta = +0.00109$<br>$SE = 0.0207$<br>$t_{9285} = +0.05$<br>$p = 0.96$
N_{obs}	9301	9301
$adj-R^2$	0.79	0.79
BIC	14211.05	14502.83

Table S5. Fixed effects of the analysis of the relationship between questionnaire sub-scales and mean Vigour. Models were specified as follows. Model 1: vigour $\sim$ 1 + a_n + Mean RT + log(Block) + Age + Impulsivity Score*R_0 + R_0*AMI_{beh} + R_0*AMI_{emo} + R_0*AMI_{soc} + (1 + a_n + Mean RT + log(Block) + Age + Impulsivity Score*R_0 + R_0*AMI_{beh} + R_0*AMI_{emo} + R_0*AMI_{soc} | Gender) + (1 + a_n + Mean RT + R_0 + log(Block) | participant); Model 2: vigour $\sim$ 1 + a_n + Mean RT + log(Block) + Age + Impulsivity Score*R_0 + R_0*AES_{beh} + R_0*AES_{emo} + R_0*AES_{cog} + R_0*$\text{AES}_{\text{other}}$ + (1 + a_n + Mean RT + log(Block) + Age + Impulsivity Score*R_0 + R_0*AES_{beh} + R_0*AES_{emo} + R_0*AES_{cog} + R_0*$\text{AES}_{\text{other}}$ | Gender) + (1 + a_n + Mean RT + R_0 + log(Block) | participant).

	Model 1	Model 2
(Intercept)	$\beta = -0.0103$<br>$SE = 0.0735$<br>$t_{475} = -0.14$<br>$p = 0.89$	$\beta = -0.0199$<br>$SE = 0.0715$<br>$t_{466} = -0.28$<br>$p = 0.78$
Time-on-task	$\beta = +0.336$<br>$SE = 0.0558$<br>$t_{475} = +6.02$<br>***p<0.0001***	$\beta = +0.327$<br>$SE = 0.0535$<br>$t_{466} = +6.10$<br>***p<0.0001***
Baseline Fatigue	$\beta = +0.833$<br>$SE = 0.0468$<br>$t_{475} = +17.79$<br>***p<0.0001***	$\beta = +0.823$<br>$SE = 0.0855$<br>$t_{466} = +9.63$<br>***p<0.0001***
Block Reward	$\beta = -0.0698$<br>$SE = 0.0261$<br>$t_{475} = -2.68$<br>***p = 0.0076***	$\beta = -0.068$<br>$SE = 0.0279$<br>$t_{466} = -2.43$<br>***p = 0.015***
Block Vigour	$\beta = +0.0803$<br>$SE = 0.0345$<br>$t_{475} = +2.33$<br>***p = 0.02***	$\beta = +0.074$<br>$SE = 0.0389$<br>$t_{466} = +1.90$<br>$p = 0.06$
age		$\beta = -0.012$<br>$SE = 0.051$<br>$t_{466} = -0.24$<br>$p = 0.81$
Apathy Score		$\beta = -0.1$<br>$SE = 0.108$<br>$t_{466} = -0.93$<br>$p = 0.35$
Apathy Score:Time-on-task		$\beta = -0.0977$<br>$SE = 0.0786$<br>$t_{466} = -1.24$<br>$p = 0.21$
Impulsivity Score		$\beta = +0.0435$<br>$SE = 0.0918$<br>$t_{466} = +0.47$<br>$p = 0.64$
Impulsivity Score:Time-on-task		$\beta = +0.0151$<br>$SE = 0.072$<br>$t_{466} = +0.21$<br>$p = 0.83$
Block Reward:Apathy Score		$\beta = +0.0821$<br>$SE = 0.0427$<br>$t_{466} = +1.92$<br>$p = 0.06$
Block Reward:Impulsivity Score		$\beta = -0.007$<br>$SE = 0.039$<br>$t_{466} = -0.18$<br>$p = 0.86$
Block Vigour:Apathy Score		$\beta = -0.039$<br>$SE = 0.0456$<br>$t_{466} = -0.86$<br>$p = 0.39$
Block Vigour:Impulsivity Score		$\beta = +0.0357$<br>$SE = 0.051$<br>$t_{466} = +0.70$<br>$p = 0.48$
N_{obs}	480	480
$adj-R^2$	0.97	0.97
BIC	356.54	1037.46

Table S6. Fixed effects of the analysis of fatigue ratings. Models were specified as follows. Model 1: fatigue $\sim$ 1 + Block Reward + Block Vigour + Baseline Fatigue + Time-on-task + (1 + Block Reward + Block Vigour + Time-on-task | participant); Model 2: fatigue $\sim$ 1 + age + Baseline Fatigue + Block Reward*Apathy Score + Block Vigour*Apathy Score + Block Reward*Impulsivity Score + Block Vigour*Impulsivity Score + Apathy Score*Time-on-task + Impulsivity Score*Time-on-task + (1 + age + Baseline Fatigue + Block Reward*Apathy Score + Block Vigour*Apathy Score + Block Reward*Impulsivity Score + Block Vigour*Impulsivity Score + Apathy Score*Time-on-task + Impulsivity Score*Time-on-task | female) + (1 + Block Reward + Block Vigour + Time-on-task | participant).

	Model 1	Model 2
(Intercept)	$\beta = -0.00464$<br>$SE = 0.0669$<br>$t_{466} = -0.07$<br>$p = 0.94$	$\beta = -0.0206$<br>$SE = 0.0705$<br>$t_{464} = -0.29$<br>$p = 0.77$
Time-on-task	$\beta = +0.33$<br>$SE = 0.056$<br>$t_{466} = +5.90$<br>***p<0.0001***	$\beta = +0.324$<br>$SE = 0.0575$<br>$t_{464} = +5.64$<br>***p<0.0001***
age	$\beta = +0.0309$<br>$SE = 0.0464$<br>$t_{466} = +0.67$<br>$p = 0.51$	$\beta = -0.0601$<br>$SE = 0.0377$<br>$t_{464} = -1.59$<br>$p = 0.11$
Baseline Fatigue	$\beta = +0.807$<br>$SE = 0.0616$<br>$t_{466} = +13.11$<br>***p<0.0001***	$\beta = +0.842$<br>$SE = 0.0832$<br>$t_{464} = +10.12$<br>***p<0.0001***
Impulsivity Score	$\beta = -0.0134$<br>$SE = 0.0484$<br>$t_{466} = -0.28$<br>$p = 0.78$	$\beta = +0.0256$<br>$SE = 0.0829$<br>$t_{464} = +0.31$<br>$p = 0.76$
Block Reward	$\beta = -0.059$<br>$SE = 0.0319$<br>$t_{466} = -1.85$<br>$p = 0.07$	$\beta = -0.0777$<br>$SE = 0.0333$<br>$t_{464} = -2.33$<br>***p = 0.02***
Block Reward:Impulsivity Score	$\beta = +0.00124$<br>$SE = 0.0247$<br>$t_{466} = +0.05$<br>$p = 0.96$	$\beta = -0.0288$<br>$SE = 0.0202$<br>$t_{464} = -1.42$<br>$p = 0.16$
Block Vigour	$\beta = +0.0621$<br>$SE = 0.0358$<br>$t_{466} = +1.73$<br>$p = 0.08$	$\beta = +0.0661$<br>$SE = 0.0398$<br>$t_{464} = +1.66$<br>$p = 0.10$
AMI_{beh}	$\beta = +0.109$<br>$SE = 0.0531$<br>$t_{466} = +2.05$<br>***p = 0.041***	
AMI_{emo}	$\beta = -0.166$<br>$SE = 0.0571$<br>$t_{466} = -2.90$<br>***p = 0.0039***	
AMI_{soc}	$\beta = +0.0411$<br>$SE = 0.0566$<br>$t_{466} = +0.73$<br>$p = 0.47$	
Block Reward:AMI_{beh}	$\beta = +0.0508$<br>$SE = 0.0251$<br>$t_{466} = +2.03$<br>***p = 0.043***	
Block Reward:AMI_{emo}	$\beta = -0.00239$<br>$SE = 0.0407$<br>$t_{466} = -0.06$<br>$p = 0.95$	
Block Reward:AMI_{soc}	$\beta = +0.011$<br>$SE = 0.0208$<br>$t_{466} = +0.53$<br>$p = 0.60$	
AES_{beh}		$\beta = -0.0516$<br>$SE = 0.0917$<br>$t_{464} = -0.56$<br>$p = 0.57$
AES_{cog}		$\beta = -0.134$<br>$SE = 0.0613$<br>$t_{464} = -2.19$<br>***p = 0.029***
AES_{emo}		$\beta = -0.0417$<br>$SE = 0.052$<br>$t_{464} = -0.80$<br>$p = 0.42$
AES_{other}		$\beta = +0.239$<br>$SE = 0.0541$<br>$t_{464} = +4.41$<br>***p<0.0001***
Block Reward:AES_{beh}		$\beta = +0.0102$<br>$SE = 0.0351$<br>$t_{464} = +0.29$<br>$p = 0.77$
Block Reward:AES_{cog}		$\beta = +0.0485$<br>$SE = 0.0338$<br>$t_{464} = +1.44$<br>$p = 0.15$
Block Reward:AES_{emo}		$\beta = -0.0432$<br>$SE = 0.0339$<br>$t_{464} = -1.27$<br>$p = 0.20$
Block Reward:AES_{other}		$\beta = +0.0387$<br>$SE = 0.0873$<br>$t_{464} = +0.44$<br>$p = 0.66$
N_{obs}	480	480
$adj-R^2$	0.97	0.97
BIC	1031.01	1204.70

Table S7. Fixed effects of the analysis of the relationship between questionnaire sub-scales and fatigue ratings. Models were specified as follows. Model 1: fatigue $\sim$ 1 + Block Vigour + age + Baseline Fatigue + Time-on-task + Block Reward*AMI_{emo} + Block Reward*AMI_{soc} + Block Reward*AMI_{beh} + Block Reward*Impulsivity Score + (1 + Block Vigour + age + Baseline Fatigue + Time-on-task + Block Reward*AMI_{emo} + Block Reward*AMI_{soc} + Block Reward*AMI_{beh} + Block Reward*Impulsivity Score | female) + (1 + Block Reward + Block Vigour + Time-on-task | participant); Model 2: fatigue $\sim$ 1 + Block Vigour + age + Baseline Fatigue + Time-on-task + Block Reward*AES_{cog} + Block Reward*AES_{beh} + Block Reward*AES_{emo} + Block Reward*AES_{other} + Block Reward*Impulsivity Score + (1 + Block Vigour + age + Baseline Fatigue + Time-on-task + Block Reward*AES_{cog} + Block Reward*AES_{beh} + Block Reward*AES_{emo} + Block Reward*AES_{other} + Block Reward*Impulsivity Score | female) + (1 + Block Reward + Block Vigour + Time-on-task | participant).

	Model 1
(Intercept)	$\beta = +0.418$<br>$SE = 0.00506$<br>$t_{56} = +82.56$<br>***p<0.0001***
Age	$\beta = +0.0108$<br>$SE = 0.00516$<br>$t_{56} = +2.08$<br>***p = 0.042***
Apathy Score	$\beta = +0.00226$<br>$SE = 0.00542$<br>$t_{56} = +0.42$<br>$p = 0.68$
Impulsivity Score	$\beta = +0.00468$<br>$SE = 0.00547$<br>$t_{56} = +0.86$<br>$p = 0.40$
N_{obs}	60
$adj - R^2$	0.03
BIC	-156.98

Table S8. Fixed effects of the analysis of mean reaction time on the Reactive Control Task. The model was specified as follows: RT $\sim$ 1 + Age + Apathy Score + Impulsivity Score + (1 + Age + Apathy Score + Impulsivity Score | Gender).

	Model 1	Model 2
(Intercept)	$\beta = +8.09 \times 10^{-16}$<br>$SE = 0.114$<br>$t_{478} = +0.00$<br>$p = 1.00$	$\beta = +0.0448$<br>$SE = 0.149$<br>$t_{472} = +0.30$<br>$p = 0.76$
log(Block)	$\beta = -0.0979$<br>$SE = 0.0341$<br>$t_{478} = -2.87$<br>***p = 0.0042***	$\beta = -0.0966$<br>$SE = 0.031$<br>$t_{472} = -3.11$<br>***p = 0.002***
Age		$\beta = -0.0753$<br>$SE = 0.172$<br>$t_{472} = -0.44$<br>$p = 0.66$
Apathy Score		$\beta = +0.0922$<br>$SE = 0.172$<br>$t_{472} = +0.54$<br>$p = 0.59$
Impulsivity Score		$\beta = -0.0746$<br>$SE = 0.115$<br>$t_{472} = -0.65$<br>$p = 0.52$
log(Block):Age		$\beta = +0.0879$<br>$SE = 0.0359$<br>$t_{472} = +2.45$<br>***p = 0.015***
log(Block):Apathy Score		$\beta = -0.116$<br>$SE = 0.0305$<br>$t_{472} = -3.82$<br>***p = 0.00015***
log(Block):Impulsivity Score		$\beta = +0.0244$<br>$SE = 0.0446$<br>$t_{472} = +0.55$<br>$p = 0.58$
N_{obs}	480	480
$adj - R^2$	0.84	0.83
BIC	867.67	1098.05

Table S9. Fixed effects of the analysis of mean reaction time on the Predictive Control Task. Models were specified as follows. Model 1: RT $\sim$ 1 + log(Block) + (1 + log(Block) | participant); Model 2: RT $\sim$ 1 + log(Block)*Apathy Score + log(Block)*Impulsivity Score + log(Block)*Age + (1 + log(Block)*Apathy Score + log(Block)*Impulsivity Score + log(Block)*Age | Gender) + (1 + log(Block) | participant).

	Model 1	Model 2
(Intercept)	$\beta = -0.0744$<br>$SE = 0.045$<br>$t_{3838} = -1.65$<br>$p = 0.10$	$\beta = -0.0736$<br>$SE = 0.0425$<br>$t_{3834} = -1.73$<br>$p = 0.08$
Test duration	$\beta = +1.64$<br>$SE = 0.0851$<br>$t_{3838} = +19.23$<br>***p<0.0001***	$\beta = +1.64$<br>$SE = 0.0842$<br>$t_{3834} = +19.44$<br>***p<0.0001***
Apathy Score		$\beta = +0.0949$<br>$SE = 0.0356$<br>$t_{3834} = +2.67$<br>***p = 0.0076***
Apathy Score:Test duration		$\beta = -0.0678$<br>$SE = 0.0717$<br>$t_{3834} = -0.95$<br>$p = 0.34$
Impulsivity Score		$\beta = +0.0319$<br>$SE = 0.0346$<br>$t_{3834} = +0.92$<br>$p = 0.36$
Impulsivity Score:Test duration		$\beta = +0.00483$<br>$SE = 0.0685$<br>$t_{3834} = +0.07$<br>$p = 0.94$
N_{obs}	3840	3840
$adj-R^2$	0.52	0.52
BIC	3688.38	3709.53

Table S10. Fixed effects of the analysis of responses on the Temporal Duration Discrimination Task. Models were specified as follows. Model 1: saidTestIsLonger ∼ 1 + Test duration + (1 + Test duration | participant); Model 2: saidTestIsLonger ∼ 1 + Apathy Score*Test duration + Impulsivity Score*Test duration + (1 + Test duration | participant).
